# RNA Splicing of the *MsndhB* Gene and Photosynthetic Characteristics in Alfalfa (*Medicago sativa*) Under High- and Low-Temperature Stress

**DOI:** 10.3390/plants15203198

**Published:** 2026-10-18

**Authors:** Wenxian Yang, Xiaojie Zhang, Rui Wang, Xiao Han, Zhenwei Ren, Weiwei Liang, Yiqiang Ren, Lili Gu

**Affiliations:** 1 College of Grassland Science, Xinjiang Agricultural University, Urumqi 830052, China; zy1131283250@163.com (W.Y.); 18167868990@163.com (X.Z.); wr17881530650@163.com (R.W.); hx18235289246@163.com (X.H.); rzw123333@163.com (Z.R.); ren19962023@163.com (Y.R.); 2 Xinjiang Key Laboratory of Grassland Resources and Ecology, Urumqi 830052, China; 3 Key Laboratory of Grassland Resources and Ecology of Western Arid Region, Ministry of Education, Urumqi 830052, China; 4 Xinjiang Uygur Autonomous Region Academy of Animal Science, Urumqi 830011, China; caoyuanlww@126.com

**Keywords:** *Medicago sativa* L., *MsndhB*, temperature stress, RNA splicing, photosynthesis

## Abstract

RNA splicing of chloroplast-encoded genes contributes to plant responses to abiotic stress, yet its regulation remains poorly understood in forage legumes. Here, we investigated RNA splicing of the chloroplast *ndhB* gene in alfalfa (*Medicago sativa* L.) under abiotic stress and its association with photosynthetic and physiological responses under temperature stress. Using cultivar ‘Xinjiang Daye’, we characterized *MsndhB* structure and analyzed splicing efficiency across tissues, developmental stages, and four stress treatments (high temperature, low temperature, salt, and drought). Under high- and low-temperature stress, photosynthetic gas exchange, chlorophyll fluorescence, ATP, and malondialdehyde (MDA) were measured to evaluate associations with *MsndhB* splicing efficiency. The results showed that high temperature reduced splicing efficiency and caused pre-mRNA accumulation, whereas low temperature enhanced splicing; salt and drought affected transcript abundance but not splicing efficiency. Splicing efficiency was higher in green tissues and at the branching stage. Under temperature stress, splicing efficiency was correlated with photosynthetic and oxidative stress indicators, but these associations do not provide evidence of functional control. These findings suggest that *MsndhB* splicing is temperature-responsive and may be involved in chloroplast responses to temperature stress, providing a foundation for elucidating the role of post-transcriptional regulation of chloroplast genes in alfalfa responses to temperature stress.

## 1. Introduction

Alfalfa (*Medicago sativa* L.) is one of the most extensively cultivated leguminous forage crops worldwide. Owing to its high protein content, strong nitrogen-fixing capacity, and excellent palatability, alfalfa plays an important role in the global livestock industry [1]. Under natural growth conditions, alfalfa frequently encounters diverse abiotic stresses. Late spring frost events can cause substantial damage to alfalfa seedlings and plants and adversely affect growth and productivity [2,3]; salinity or high-temperature stress further suppress growth and photosynthetic capacity. With intensifying global climate change, the increasing frequency of extreme temperature events and soil salinization has severely constrained alfalfa yield and quality [4,5,6]. Moreover, substantial variations in high-temperature tolerance, photosynthetic energy-use efficiency, and cold sensitivity exist among different alfalfa cultivars [7]. Therefore, elucidating the molecular mechanisms underlying alfalfa responses to temperature stress holds significant theoretical importance and application value for breeding stress-tolerant varieties.

The chloroplast, as the primary site of photosynthesis, possesses a semi-autonomous genetic system in which gene expression is precisely regulated at both transcriptional and post-transcriptional levels [8]. Unlike nuclear genes, a significant number of chloroplast genes contain Group II introns, whose splicing is highly dependent on nuclear-encoded trans-acting factors, including RNA helicases and pentatricopeptide repeat (PPR) proteins [9,10]. In recent years, multiple splicing regulators have been identified in crops, including rice, maize, and cotton, indicating that the splicing regulatory mechanism of chloroplast Group II introns is conserved across different plant species, although the functions of specific regulators may exhibit species specificity [11,12,13,14]. In addition to the canonical PPR and CRM domain-containing proteins, chloroplast splicing proteins encompass various non-canonical RNA-binding proteins, including the RNase III domain-containing protein RNC1 and its interacting partner WTF1, which contains a plant-specific PORR domain. These two proteins can form a complex that cooperatively mediates RNA splicing [15]. Moreover, Zhao et al. identified a P-type PPR protein, WPR, in rice, which is required for the splicing of multiple group II introns including *ndhB* and recruits both splicing and editing factors to coordinate its RNA processing [16]. Among the numerous chloroplast genes that require splicing, *ndhB* encodes a core subunit of the NDH complex, and the proper processing of its transcript is essential for the production of functional mature *ndhB* mRNA [17,18].

The chloroplast NADH dehydrogenase-like (NDH) complex plays an essential role in cyclic electron flow (CEF) around photosystem I and contributes to the maintenance of photosynthetic electron transport under stress conditions [19,20]. By facilitating excess electron dissipation, balancing ATP and NADPH production, and preventing excessive stromal reduction, the NDH complex helps maintain chloroplast redox homeostasis. The NDH complex consists of multiple chloroplast- and nuclear-encoded subunits, among which *ndhB* encodes a membrane protein of the NDH complex [20]. Recent studies have shown that the NDH-PSI supercomplex provides a structural framework for efficient CEF in vascular plants and the stability of the PSI-NDH supercomplex is important for maintaining NDH activity and protecting photosynthetic electron transport under chilling stress [21,22]. In addition, NDH-mediated CEF has been shown to contribute to the maintenance of photosynthetic performance and biomass production in rice under low-temperature conditions (20 °C), suggesting its involvement in low-temperature adaptation [23,24]. In several plant species, the *ndhB* transcript undergoes RNA splicing, and this process is affected by temperature changes. For example, high temperature reduces *ndhB* splicing efficiency in tobacco [18], whereas CDE4 is required for maintaining normal *ndhB* splicing under low-temperature conditions in rice [25]. Similarly, the *Arabidopsis* AtRH3 mutant shows reduced *ndhB* splicing efficiency during low-temperature stress [26]. However, in alfalfa, one of the most widely cultivated forage legumes, the splicing of the chloroplast *ndhB* transcript remains uncharacterized. It is unknown whether *MsndhB* RNA splicing responds to temperature stress in a manner similar to that in tobacco, rice and *Arabidopsis* or exhibits species-specific regulation, and whether its splicing efficiency is associated with photosynthetic performance under stress.

Moreover, salt and drought are among the major abiotic stresses limiting alfalfa production. Whether chloroplast *ndhB* splicing is also responsive to these stresses, or whether its responsiveness is restricted to temperature, remains unknown. In this study, we cloned the *MsndhB* gene from the alfalfa cultivar ‘Xinjiang Daye’ and characterized its structural features. Using endpoint RT-PCR and qRT-PCR, we systematically evaluated *MsndhB* RNA splicing efficiency under high-temperature, low-temperature, salt and drought stresses, as well as across different developmental stages and tissue types. Meanwhile, photosynthetic gas exchange, chlorophyll fluorescence and related physiological indicators were measured under temperature stress to assess their association with *MsndhB* RNA splicing efficiency. These analyses aimed to elucidate the temperature responsiveness and stress specificity of *MsndhB* RNA splicing and to provide insights into the post-transcriptional regulation of chloroplast genes during stress adaptation in forage plants.

## 2. Results

### 2.1. Structural Characterization and Conservation Analysis of MsndhB

**Figure 1:**
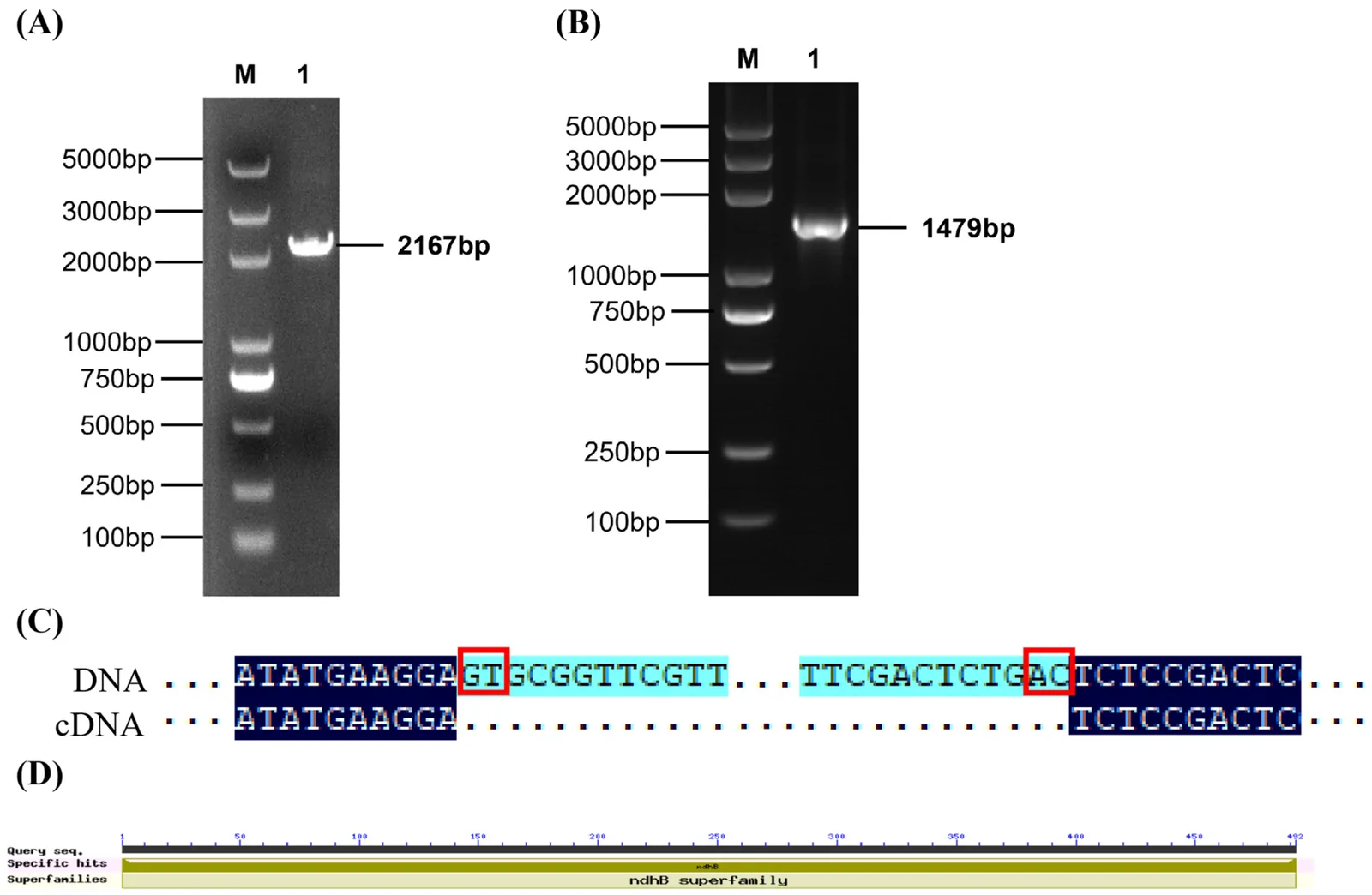
Cloning and Characterization of the *MsndhB* Gene and Conserved Domain Analysis of the MsNDHB Protein in ‘Xinjiang Daye’ Alfalfa (*Medicago sativa*). (**A**) *MsndhB* DNA sequence; M: DL5000. 1: *MsndhB* DNA amplified fragment; (**B**) *MsndhB* cDNA sequence. M: DL5000; 1: *MsndhB* cDNA amplified fragment. (**C**) 5′and 3′ splice sites; (**D**) Conserved domains of the MsNDHB protein.

Sequencing results showed that the *MsndhB* gene from *Medicago sativa* ‘Xinjiang Daye’ was 2167 bp in length, comprising a 1479 bp coding sequence (CDS) and a 688 bp Group II intron (Figure 1A,B). The cloned *MsndhB* sequence has been deposited in the NCBI GenBank database under accession number [QB044729]. Sequence alignment showed that *MsndhB* differed from the reference genome sequence at 12 nucleotide positions (8 in exons and 4 in the intron) (Figure S1). Of the eight exonic substitutions, six were non-synonymous and two were synonymous. At the protein level, MsNDHB shared 98.78% amino acid identity with the reference sequence (NC_042841.1). The intron possessed canonical 5′-GT and 3′-AC splice sites (Figure 1C,D). According to the conserved domain analysis, the MsNDHB protein belonged to the NDHB superfamily and contained a NADH dehydrogenase subunit 2 domain (492 aa) (Figure 1D); its physicochemical properties and amino acid composition were presented in Supplementary Tables S3 and S4.

**Figure 2:**
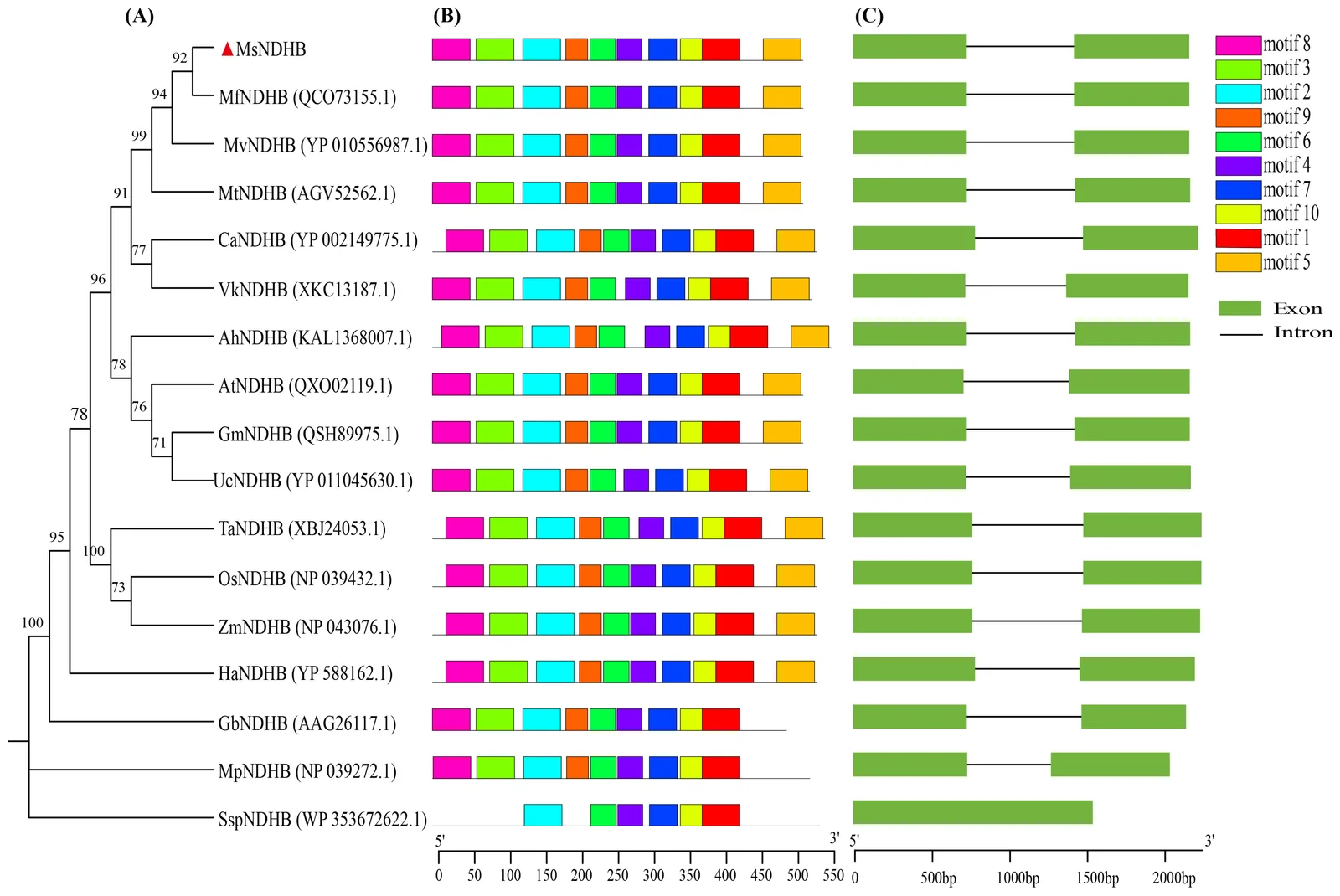
Phylogenetic analysis, conserved motifs, and gene structure of NDH-B proteins. (**A**) Phylogenetic tree of NDH-B proteins. (**B**) Conserved motif distribution in NDH-B proteins. (**C**) Gene structure of *ndhB*. The red triangle indicates *MsndhB* from *Medicago sativa* cv. Xinjiang Daye. Abbreviations: Mf., *Medicago falcata*; Mv., *Medicago varia*; Mt., *Medicago truncatula*; Ca., *Cicer arietinum*; Vk., *Vicia kulingana*; Ah., *Arachis hypogaea*; At., *Arabidopsis thaliana*; Gm., *Glycine max*; Uc., *Uraria crinita*; Ta., *Triticum aestivum*; Os., *Oryza sativa*; Zm., *Zea mays*; Ha., *Helianthus annuus*; Gb., *Ginkgo biloba*; Mp., *Marchantia polymorpha*; Ssp., *Synechocystis* sp.

The amino acid sequences of the 17 NDHB proteins used for the phylogenetic analysis are provided in Supplementary Table S7. Phylogenetic analysis showed that MsNDHB of *Medicago sativa* ‘Xinjiang Daye’ clustered with NDHB proteins of other *Medicago* species, including *M. falcata* and *M. truncatula*, forming a well-supported clade (Figure 2A). This clade was closely related to other leguminous genera within Fabaceae, whereas *Arabidopsis* and grass species occupied more distant positions in the phylogenetic tree. Motif analysis showed that NDHB proteins from angiosperms shared similar conserved motif patterns. The terminal motif 5 was absent in *Ginkgo* and *Marchantia*, while cyanobacterial NDHB proteins lacked several motifs conserved in higher plants. Gene structure analysis revealed that *ndhB* genes in land plants generally contained two exons and one intron, whereas cyanobacterial *ndhB* genes lacked introns (Figure 2B,C).

### 2.2. Structural Prediction of NDHB and Evolutionary Conservation of ndhB RNA Splicing Features

**Figure 3:**
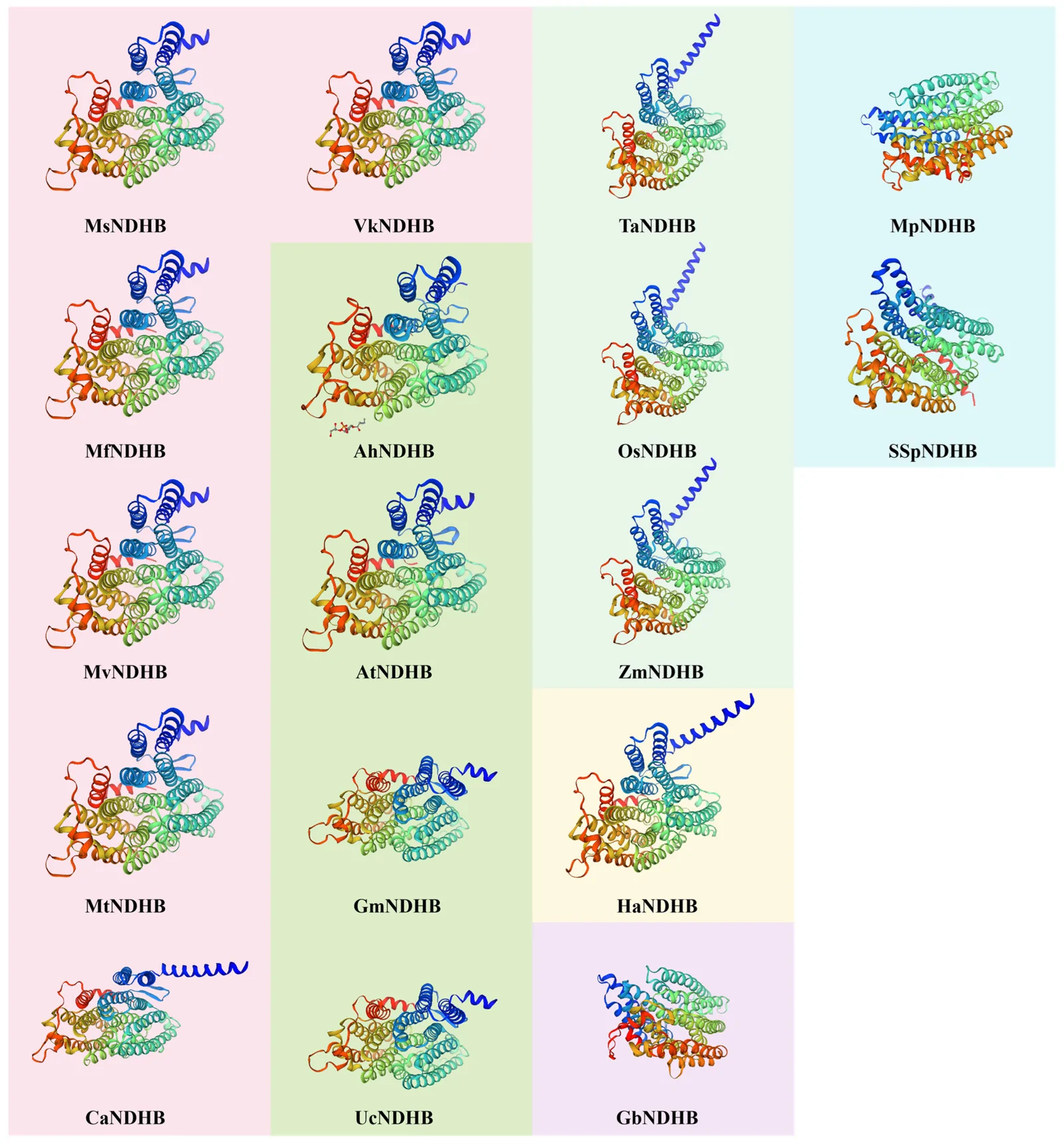
Predicted tertiary structures of NDH-B proteins from different species. Background colors group proteins by phylogenetic affinity (see Figure 2A).

Protein structure prediction revealed that NDHB homologous proteins from different species contained four typical secondary structure elements and exhibited high conservation, with α-helices being the predominant secondary structure component (Table S5). Homology models of the 17 NDHB proteins were constructed using SWISS-MODEL, and the corresponding model parameters are summarized in Table S6 (sequence identity 80.30–100%; coverage 95–100%; GMQE 0.85–0.93). Model quality was further evaluated with MolProbity, with 89.33–98.29% of the residues located in Ramachandran-favoured regions and MolProbity scores of 0.89–1.63 (Table S6), indicating that the predicted structures were of good quality. However, because most templates used were AlphaFold-derived structures, the predicted models should be interpreted as computational models that require experimental validation. Moreover, the secondary structures predicted from the amino acid sequences (Table S5) agreed well with the three-dimensional models shown in Figure 3.

Analysis of *ndhB* splice sites among different species revealed that *ndhB* introns in most land plants contained canonical 5′-GT/3′-AC Group II splice boundaries, whereas the 3′ splice site varied in *Uraria crinita* and *Vicia kulingana*, the latter additionally exhibiting a non-canonical 5′-GA splice site (Table 1). These findings indicate that the RNA splicing characteristics of *ndhB* are highly conserved in higher plants, thus laying the foundation for subsequent investigations into the changes in RNA splicing patterns of *MsndhB* under abiotic stresses.

### 2.3. Changes in RNA Splicing of the MsndhB Gene Under Different Abiotic Stresses

**Figure 4:**
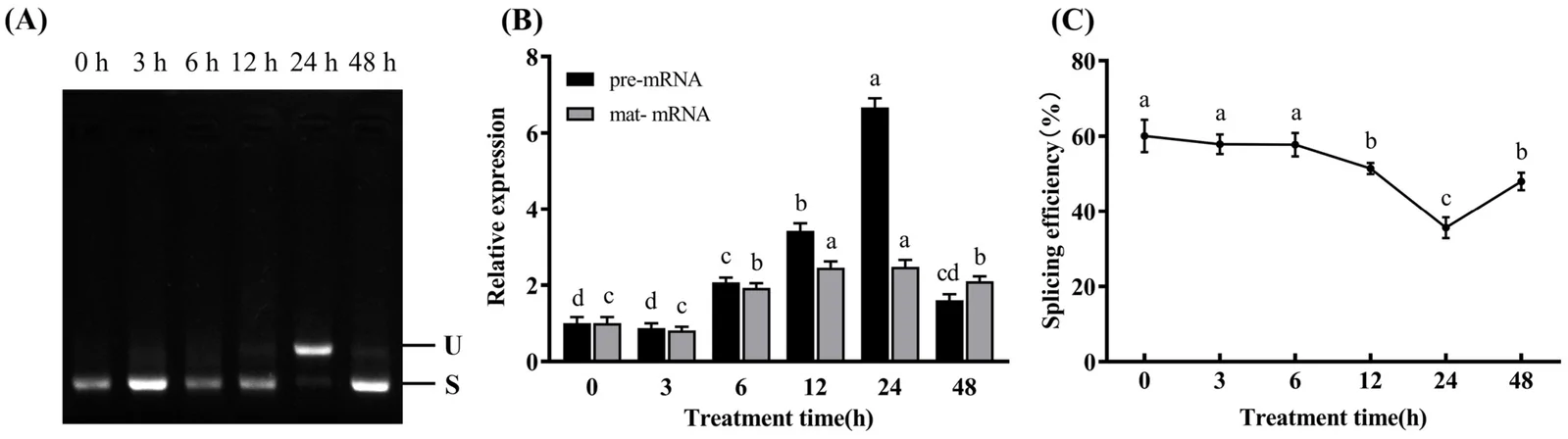
Changes in RNA splicing of the *MsndhB* gene under high-temperature stress in *Medicago sativa* ‘Xinjiang Daye’. Thirty-day-old hydroponically grown seedlings were exposed to 38 °C under a 16 h light/8 h dark photoperiod for 0, 3, 6, 12, 24, and 48 h. The second and third fully expanded leaves from the top were harvested for RNA extraction. (**A**) Representative endpoint RT-PCR of *MsndhB* pre-mRNA (U, 1910 bp) and mature mRNA (S, 1222 bp); shown only to verify amplicon identity, not for quantification. (**B**) Relative pre-mRNA and mature mRNA levels determined by qRT-PCR with *MsActin* as the reference gene. (**C**) *MsndhB* RNA splicing efficiency. Values are presented as means ± SD of three independent experiments (*n* = 3). In panel (**B**), different lowercase letters above the bars indicate significant differences among treatment times within the same transcript type; in panel (**C**), different lowercase letters above the data points indicate significant differences in splicing efficiency among treatment times (*p* ≤ 0.05; one-way ANOVA followed by Tukey’s multiple comparison test).

Endpoint RT-PCR was used to verify the presence and sizes of *MsndhB* pre-mRNA and mature mRNA. The unspliced product was weakly detected at the early time points but became most prominent at 24 h and declined again at 48 h, whereas the spliced product was detected at all time points (Figure 4A). Because endpoint RT-PCR is not strictly quantitative, the sudden intensification of certain bands in Figure 4A should not be interpreted as an abrupt change in transcript abundance; the temporal changes in transcript abundance were quantitatively assessed by qRT-PCR (Figure 4B).

Splicing efficiency analysis and qRT-PCR results (Figure 4B,C) showed that *MsndhB* splicing efficiency remained relatively stable at approximately 60% during the first 6 h of high-temperature treatment. At 12 h, the relative transcript levels of both pre-mRNA and mature mRNA increased, whereas splicing efficiency began to decline. At 24 h, pre-mRNA abundance reached its highest level, while mature mRNA levels remained relatively stable, corresponding to the lowest splicing efficiency. At 48 h, pre-mRNA abundance decreased and splicing efficiency partially recovered; however, it remained significantly lower than that of the untreated control.

These results demonstrate that high-temperature stress significantly inhibited RNA splicing efficiency of the *MsndhB* gene, accompanied by a substantial accumulation of precursor mRNA. The inhibitory effect was most pronounced at 24 h, followed by a partial recovery of splicing efficiency at the later stage of stress.

**Figure 5:**
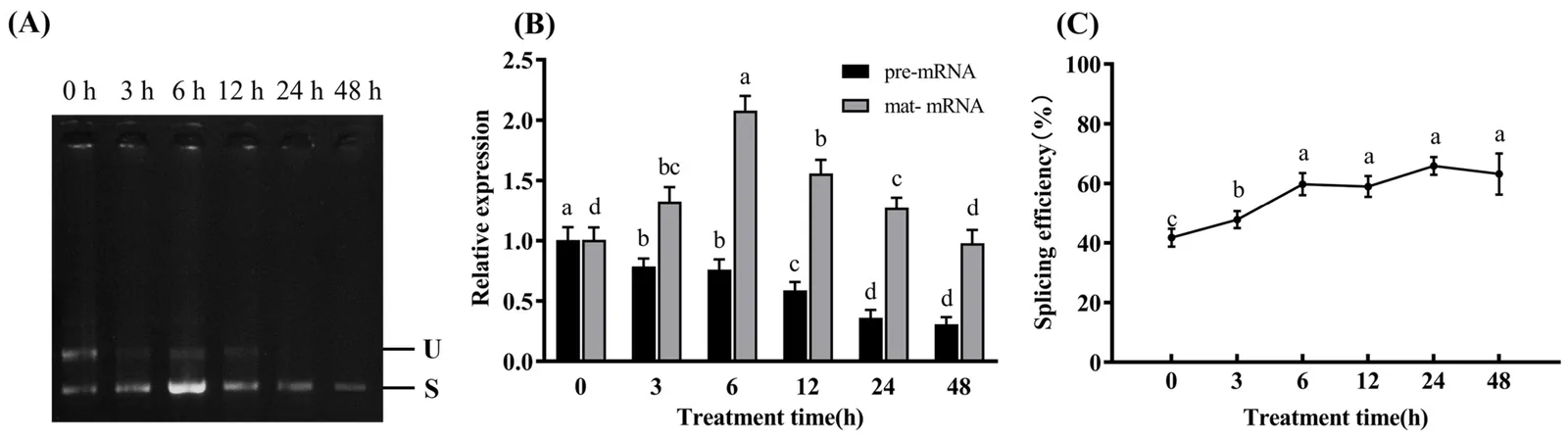
Changes in RNA splicing of the *MsndhB* gene under low-temperature stress in *Medicago sativa* ‘Xinjiang Daye’. Thirty-day-old hydroponically grown seedlings were exposed to 4 °C under a 16 h light/8 h dark photoperiod for 0, 3, 6, 12, 24, and 48 h. The second and third fully expanded leaves from the top were harvested for RNA extraction. (**A**) Representative endpoint RT-PCR of *MsndhB* pre-mRNA (U, 1910 bp) and mature mRNA (S, 1222 bp); shown only to verify amplicon identity, not for quantification. (**B**) Relative pre-mRNA and mature mRNA levels determined by qRT-PCR with *MsActin* as the reference gene. (**C**) *MsndhB* RNA splicing efficiency. Values are presented as means ± SD of three independent experiments (*n* = 3). In panel (**B**), different lowercase letters above the bars indicate significant differences among treatment times within the same transcript type; in panel (**C**), different lowercase letters above the data points indicate significant differences in splicing efficiency among treatment times (*p* ≤ 0.05; one-way ANOVA followed by Tukey’s multiple comparison test).

Endpoint RT-PCR was used to verify the presence and sizes of *MsndhB* pre-mRNA and mature mRNA under low-temperature stress. The unspliced product was clearly detected at 0–12 h but became much weaker at 24 h and was almost undetectable at 48 h, whereas the spliced product was detected at all time points and was most prominent at 6 h (Figure 5A). Because endpoint RT-PCR is not strictly quantitative, band intensities in Figure 5A were not interpreted as precise quantitative changes; the expression kinetics were evaluated by qRT-PCR (Figure 5B).

qRT-PCR and splicing efficiency analyses (Figure 5B,C) revealed that at 3–6 h, the relative transcript abundance of pre-mRNA was downregulated, whereas the relative transcript abundance of mature mRNA was significantly increased, accompanied by a rapid enhancement of splicing efficiency. The mature mRNA’s relative transcript abundance peaked at 6 h. Subsequently, the relative transcript abundances of both pre-mRNA and mature mRNA continued to decrease, while splicing efficiency reached its maximum at 24 h (approximately 63%). At 48 h, splicing efficiency slightly decreased, but it remained at a relatively high level.

In summary, these results indicated that low-temperature stress significantly enhanced *MsndhB* RNA splicing efficiency, accompanied by a gradual reduction in pre-mRNA abundance and an initial increase followed by a decrease in the relative transcript abundance of mature mRNA. Splicing efficiency increased markedly at 6 h and remained at a high level thereafter, reaching its maximum at 24 h.

**Figure 6:**
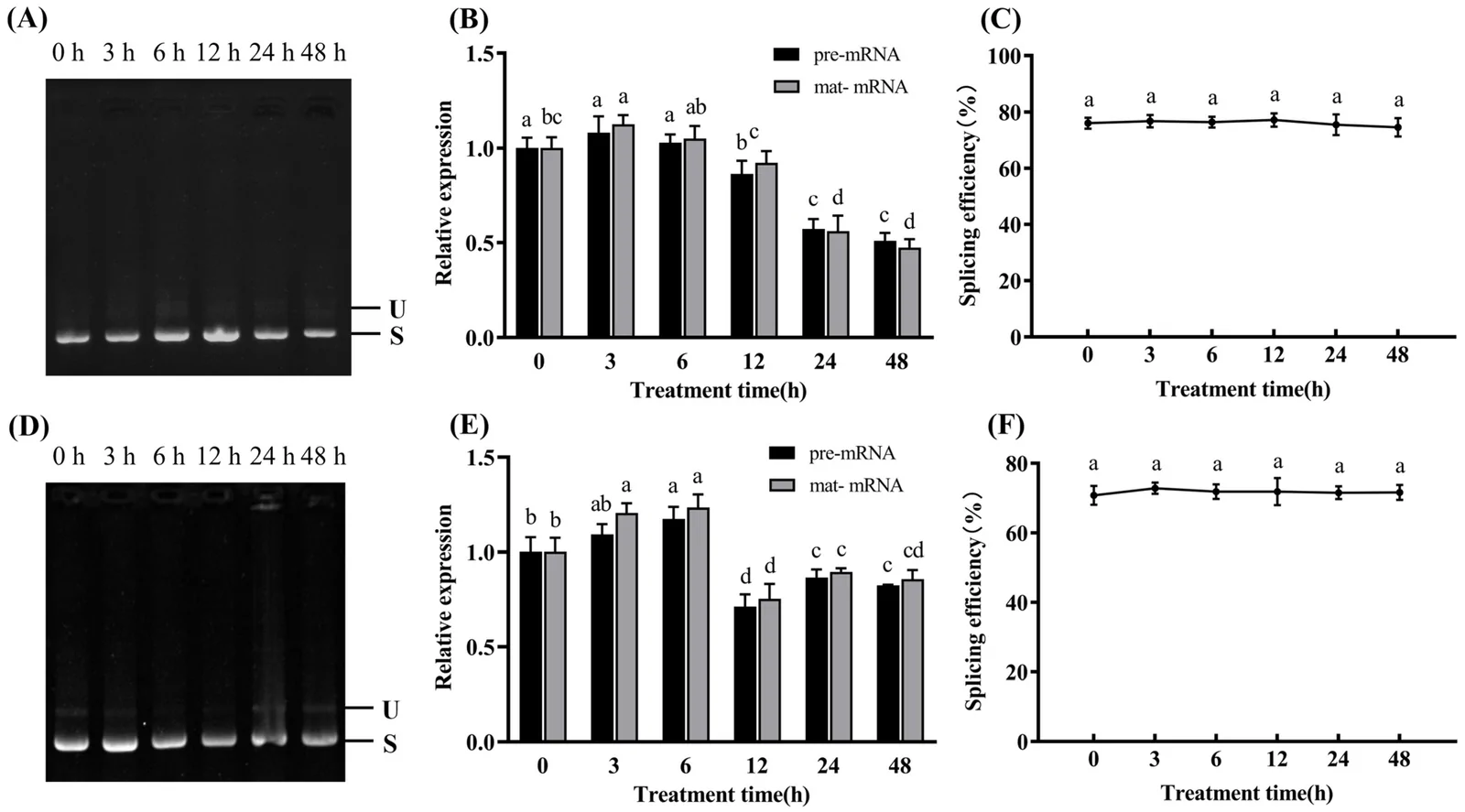
Changes in RNA splicing of the *MsndhB* gene under salt and drought stresses in *Medicago sativa* ‘Xinjiang Daye’. Thirty-day-old hydroponically grown seedlings were subjected to salt stress (200 mM NaCl) or drought stress (20% PEG-6000) for 0, 3, 6, 12, 24, and 48 h. The second and third fully expanded leaves from the top were harvested for RNA extraction. (**A**,**D**) Representative endpoint RT-PCR of *MsndhB* pre-mRNA (U, 1910 bp) and mature mRNA (S, 1222 bp) under salt (**A**) and drought (**D**) stresses; shown only to verify amplicon identity, not for quantification. (**B**,**E**) Relative pre-mRNA and mature mRNA levels determined by qRT-PCR with *MsActin* as the reference gene under salt (**B**) and drought (**E**) stresses. (**C**,**F**) *MsndhB* RNA splicing efficiency under salt (**C**) and drought (**F**) stresses. Values are presented as means ± SD of three independent experiments (*n* = 3). In panels (**B**,**E**), different lowercase letters above the bars indicate significant differences among treatment times within the same transcript type; in panels (**C**,**F**), different lowercase letters above the data points indicate significant differences in splicing efficiency among treatment times (*p* ≤ 0.05; one-way ANOVA followed by Tukey’s multiple comparison test).

Endpoint RT-PCR results (Figure 6A,D) demonstrated that under salt and drought stresses, the pre-mRNA and mature mRNA bands remained relatively stable at all time points, with no obvious changes being observed. Because endpoint RT-PCR is not strictly quantitative, band intensities in Figure 6A,D were not interpreted as precise quantitative changes; the expression kinetics were evaluated by qRT-PCR (Figure 6B,E).

qRT-PCR and splicing efficiency analyses (Figure 6B,C,E,F) revealed that splicing efficiency remained relatively stable under both stress treatments (*p* > 0.05), whereas the relative transcript abundances of *MsndhB* exhibited significant variations (*p* ≤ 0.05). Under salt stress, the relative abundances of both pre-mRNA and mature mRNA gradually decreased over the treatment period. Initially, the relative abundances of pre-mRNA and mature mRNA increased under drought stress, reached a minimum at 12 h, and then began to gradually increase again. To confirm that the salt and drought treatments induced physiological stress responses, photosynthetic gas exchange, chlorophyll fluorescence and the contents of chlorophyll, ATP and MDA were measured. Under both salt and drought stresses, Pn, Gs, Ci, E, Fv/Fm, ATP content and total chlorophyll content decreased, whereas MDA content increased significantly (Figures S2 and S3), confirming that the applied stress treatments were effective.

Collectively, these findings suggest that neither salt nor drought stress significantly affected the RNA splicing efficiency of the *MsndhB* gene; however, both stresses did affect the overall transcript abundance of *MsndhB*.

### 2.4. Developmental Stage- and Tissue-Specific Changes in MsndhB RNA Splicing

**Figure 7:**
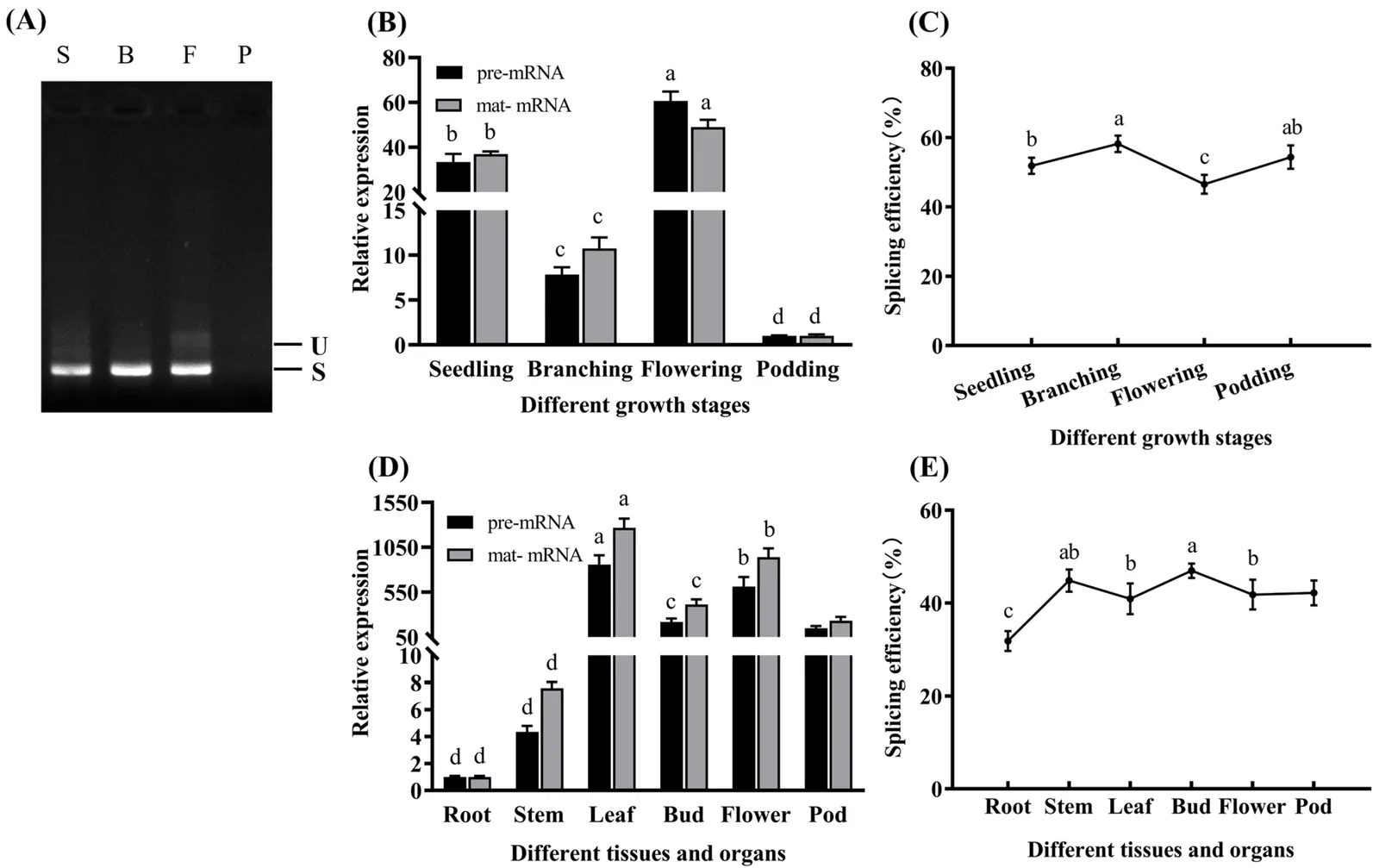
Changes in RNA splicing of the *MsndhB* gene across different growth stages and in different tissues of *Medicago sativa* ‘Xinjiang Daye’. Plants were soil-grown. Apical leaves were collected at the seedling (S), branching (B), flowering (F), and pod-setting (P) stages. At the early flowering stage, roots, stems, leaves, flower buds, and flowers were collected; pods were collected at the pod-setting stage. (**A**) Representative endpoint RT-PCR of *MsndhB* pre-mRNA (U, 1910 bp) and mature mRNA (S, 1222 bp); this panel verifies amplicon identity and was not used for quantification. (**B**,**C**) Relative pre-mRNA and mature mRNA levels (**B**) and splicing efficiency (**C**) across growth stages. (**D**,**E**) Relative pre-mRNA and mature mRNA levels (**D**) and splicing efficiency (**E**) across tissues. Relative expression was determined by qRT-PCR with *MsActin* as the reference gene. Values are means ± SD (*n* = 3). Different lowercase letters indicate significant differences (*p* ≤ 0.05; one-way ANOVA followed by Tukey’s test). Pods were excluded from the tissue comparison because they were collected at a different developmental stage.

Endpoint RT-PCR (Figure 7A) showed that the pre-mRNA band intensity at the flowering stage was markedly stronger than that at other developmental stages, whereas the mature mRNA band predominated at the seedling and branching stages.

qRT-PCR and splicing efficiency analyses (Figure 7B–E) demonstrated that across different developmental stages, the splicing efficiency during the branching stage was significantly higher than that at the flowering stage (*p* ≤ 0.05). Although the relative abundance of both pre-mRNA and mature mRNA peaked at the flowering stage, splicing efficiency was lowest at this stage. In the comparison among different tissues, stems, leaves, flower buds, and flowers exhibited significantly higher splicing effi-ciency and relative transcript abundance than roots (*p* ≤ 0.05). Pods collected at the pod-setting stage also showed high splicing efficiency and transcript abundance.

Collectively, these results indicate that *MsndhB* RNA splicing exhibits developmental stage-specific patterns and differs among tissues collected at the same developmental stage.

### 2.5. Changes in Physiological Parameters of Alfalfa Under Temperature Stress

**Figure 8:**
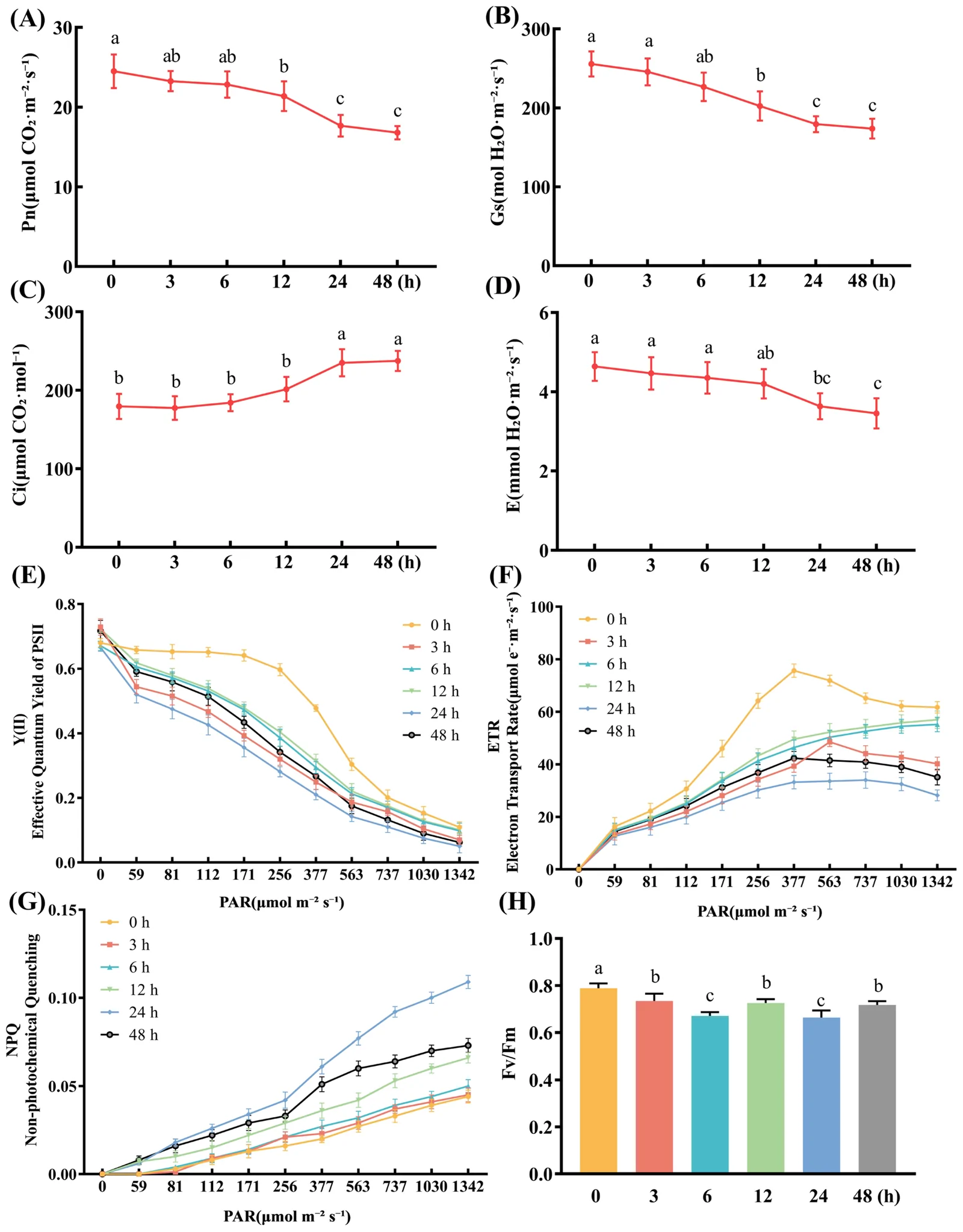
Dynamic changes in photosynthetic gas exchange and chlorophyll fluorescence parameters of *Medicago sativa* ‘Xinjiang Daye’ under high-temperature stress. Thirty-day-old seedlings grown hydroponically were exposed to high temperature at 38 °C under a 16 h light/8 h dark photoperiod for 0, 3, 6, 12, 24 and 48 h. (**A**–**D**) Pn, Gs, Ci and E, respectively. (**E**–**H**) Y(II), ETR, NPQ and Fv/Fm, respectively. Values are presented as means ± SD of three independent experiments (*n* = 3). Different lowercase letters indicate significant differences among treatment time points (*p* ≤ 0.05; one-way ANOVA followed by Tukey’s multiple comparison test).

Under high-temperature stress, Pn and Gs gradually decreased with prolonged stress duration, reaching their lowest values during 24–48 h, which were significantly lower than those of the control (*p* ≤ 0.05) (Figure 8A,B). Ci remained relatively stable during the early stage of stress, began to increase after 12 h, and peaked at 24 h (Figure 8C). E progressively decreased with stress duration, reaching its minimum at 48 h (Figure 8D).

Chlorophyll fluorescence parameters showed that Fv/Fm decreased significantly at the early stage of stress, reached its minimum at 24 h (Figure 8H), and slightly recovered at 48 h, but remained significantly lower than that of the control, indicating that the maximum photochemical efficiency of PSII was impaired. Light-response curves revealed that both Y(II) and ETR decreased with prolonged stress, whereas NPQ gradually increased (Figure 8E–G), suggesting enhanced thermal dissipation capacity.

**Figure 9:**
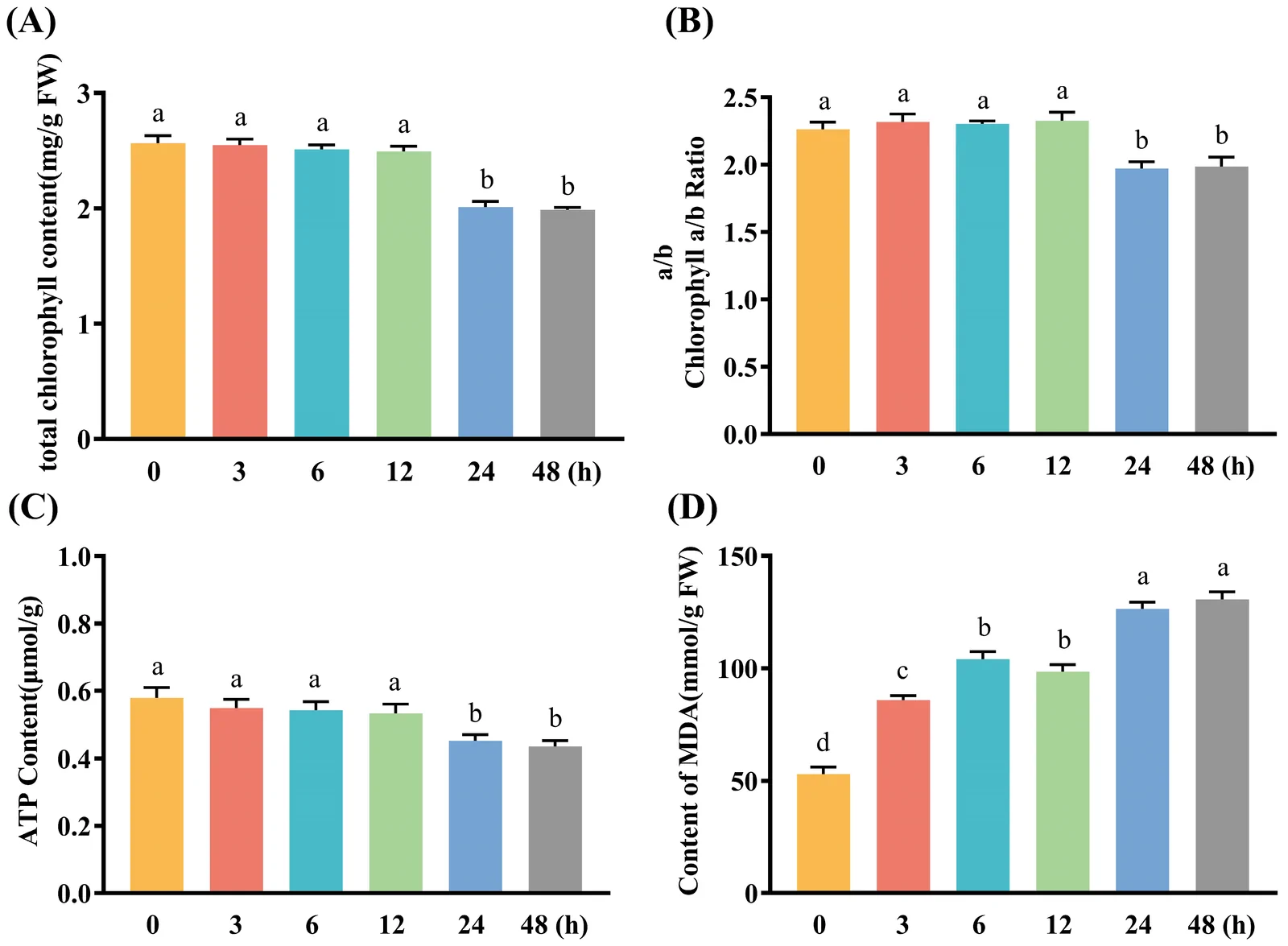
Dynamic changes in chlorophyll, ATP and MDA levels of *Medicago sativa* ‘Xinjiang Daye’ under high-temperature stress. Thirty-day-old seedlings grown hydroponically were exposed to high temperature at 38 °C under a 16 h light/8 h dark photoperiod for 0, 3, 6, 12, 24 and 48 h. (**A**–**D**) Total chlorophyll content, chlorophyll a/b ratio, ATP content and MDA content, respectively. Values are presented as means ± SD of three independent experiments (*n* = 3). Different lowercase letters indicate significant differences among treatment time points (*p* ≤ 0.05; one-way ANOVA followed by Tukey’s multiple comparison test).

Under high-temperature stress, the total photosynthetic pigment content and the Chl a/b ratio in alfalfa exhibited a continuous declining trend with treatment duration, becoming significantly lower than those of the control at 24 h (*p* ≤ 0.05) and reaching their lowest levels at 48 h (Figure 9A,B), indicating alterations in photosynthetic pigment composition. ATP content remained relatively stable before 12 h, decreased significantly at 24 h (*p* ≤ 0.05), and dropped to its minimum at 48 h (Figure 9C), suggesting that energy metabolism was potentially affected. Meanwhile, MDA content continuously accumulated with stress duration and peaked at 48 h (Figure 9D), indicating enhanced membrane lipid peroxidation.

These results indicated that under high-temperature stress, significant changes occurred in photosynthetic pigment composition, energy metabolism balance, and membrane lipid peroxidation levels in alfalfa.

**Figure 10:**
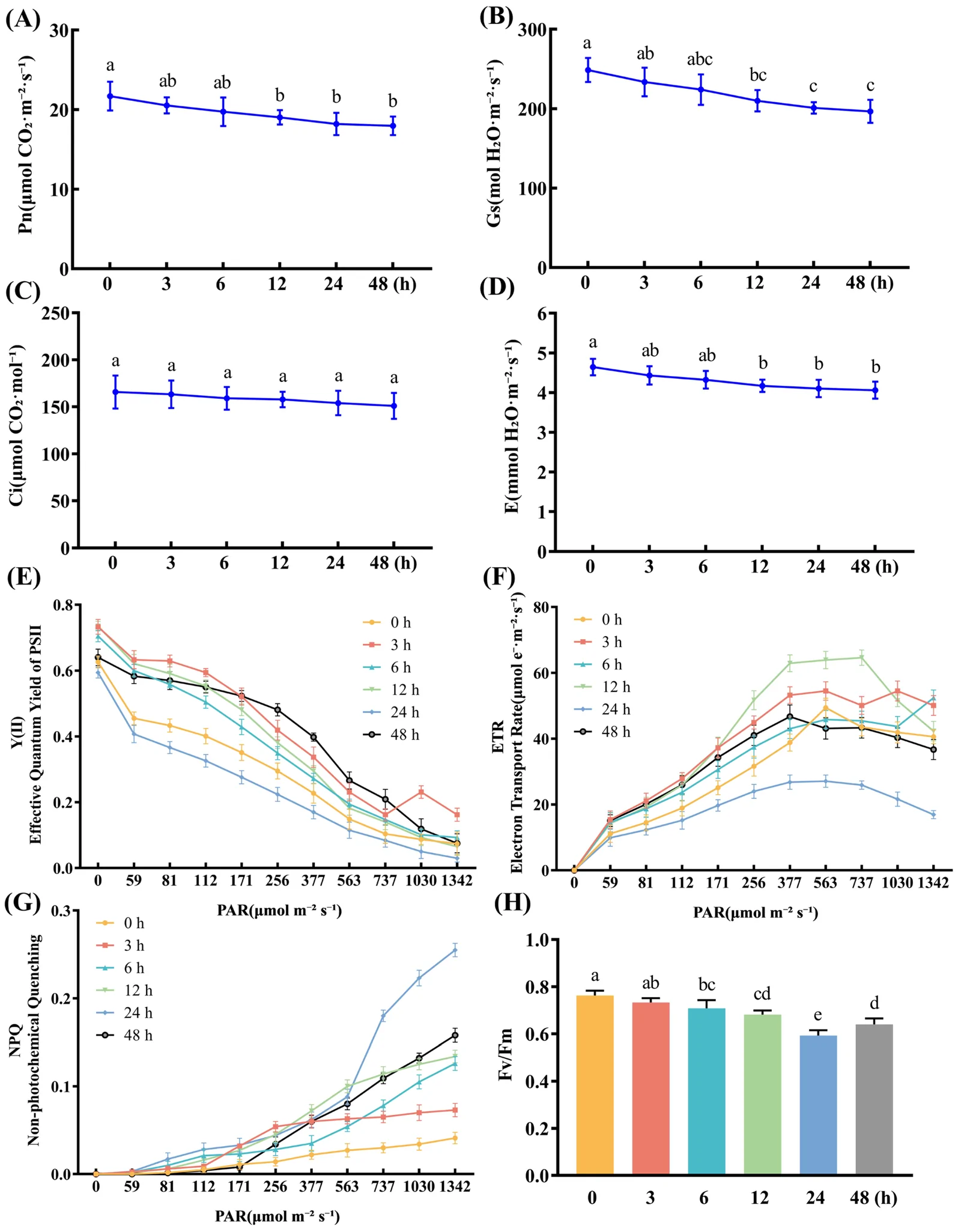
Dynamic changes in photosynthetic gas exchange and chlorophyll fluorescence parameters of *Medicago sativa* ‘Xinjiang Daye’ under low-temperature stress. Thirty-day-old seedlings grown hydroponically were exposed to low temperature at 4 °C under a 16 h light/8 h dark photoperiod for 0, 3, 6, 12, 24 and 48 h. (**A**–**D**) Pn, Gs, Ci and E, respectively. (**E**–**H**) Y(II), ETR, NPQ and Fv/Fm, respectively. Values are presented as means ± SD of three independent experiments (*n* = 3). Different lowercase letters indicate significant differences among treatment time points (*p* ≤ 0.05; one-way ANOVA followed by Tukey’s multiple comparison test).

Under low-temperature stress, Pn in alfalfa gradually decreased with prolonged treatment duration, reaching its minimum at 48 h, which was significantly lower than that of the control (Figure 10A); however, the decline was less pronounced than that under high-temperature stress. Gs and E showed continuous decreasing trends, maintaining their lowest levels at 24–48 h (Figure 10B,D). Ci remained relatively stable throughout the stress period, with no significant differences compared with the control.

Chlorophyll fluorescence parameters showed that Fv/Fm continuously declined with stress duration, reached its minimum at 24 h, and slightly recovered at 48 h, but remained significantly lower than that of the control (Figure 10H), indicating impaired PSII maximum photochemical efficiency. Light-response curves revealed that Y(II) and ETR progressively decreased with stress duration, with the most pronounced reductions observed in the 24 h and 48 h treatment groups, whereas NPQ exhibited an increasing trend (Figure 10E–G), suggesting enhanced thermal dissipation capacity under low-temperature stress.

**Figure 11:**
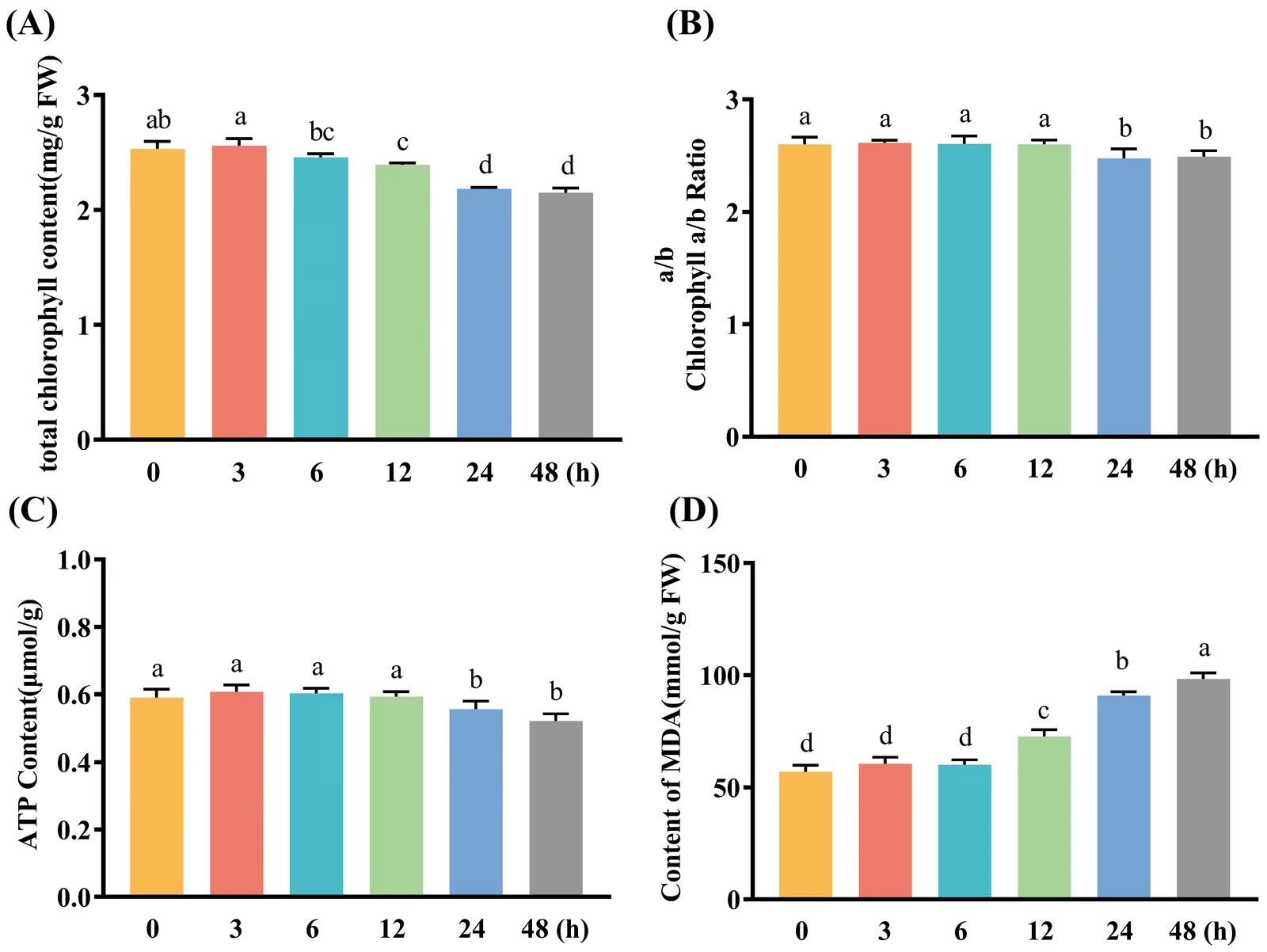
Dynamic changes in chlorophyll, ATP and MDA levels of *Medicago sativa* ‘Xinjiang Daye’ under low-temperature stress. Thirty-day-old seedlings grown hydroponically were exposed to high temperature at 38 °C under a 16 h light/8 h dark photoperiod for 0, 3, 6, 12, 24 and 48 h. (**A**–**D**) Total chlorophyll content, chlorophyll a/b ratio, ATP content and MDA content, respectively. Values are presented as means ± SD of three independent experiments (*n* = 3). Different lowercase letters indicate significant differences among treatment time points (*p* ≤ 0.05; one-way ANOVA followed by Tukey’s multiple comparison test).

Under low-temperature stress, total photosynthetic pigment content and ATP content in alfalfa continuously decreased with treatment duration, reaching their minima at 48 h. The Chl a/b ratio remained relatively stable during 0–12 h and decreased significantly at 24–48 h. MDA content continuously accumulated with stress duration and peaked at 48 h (Figure 11A–D).

Collectively, these results indicated that both low- and high-temperature stresses altered photosynthetic performance, pigment composition, energy metabolism, and oxidative balance in alfalfa, but the effects of low-temperature stress were less pronounced than those of high-temperature stress.

### 2.6. Correlation Analysis Between MsndhB Splicing Efficiency and Physiological Parameters Under Temperature Stress

**Figure 12:**
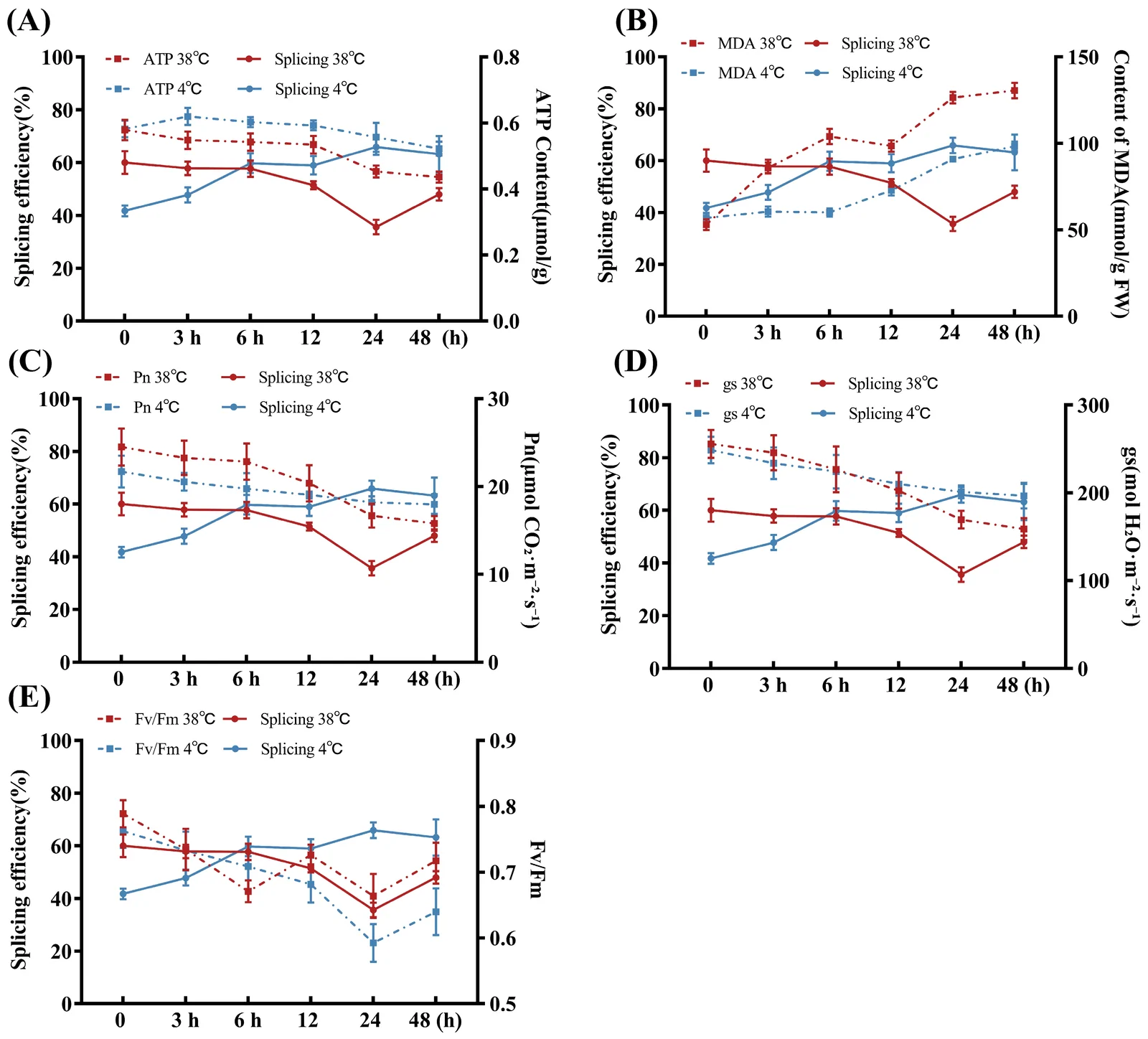
Time-series analysis of the RNA splicing efficiency of the *MsndhB* gene and key physiological indicators under temperature stress conditions. (**A**–**E**) respectively show the time-series plots of five indicators—ATP content, MDA content, Fv/Fm, Pn, and Gs—plotted against mRNA splicing efficiency of the *MsndhB* gene.

Under temperature stress, *MsndhB* RNA splicing efficiency and key physiological parameters of alfalfa exhibited time-dependent variations, with distinct response patterns observed under high- and low-temperature conditions. As shown in Figure 12, under high-temperature stress, *MsndhB* splicing efficiency reached its minimum at 24 h. During this period, ATP content, Fv/Fm, Pn, and Gs showed decreasing trends with stress duration, whereas MDA content progressively accumulated and reached its highest level at 48 h. Under low-temperature stress, *MsndhB* splicing efficiency gradually increased with stress duration, whereas ATP content, Fv/Fm, Pn, and Gs exhibited decreasing trends. Meanwhile, MDA content progressively accumulated, but its increase was much less pronounced than that under high-temperature stress, suggesting a relatively lower level of membrane lipid peroxidation.

**Figure 13:**
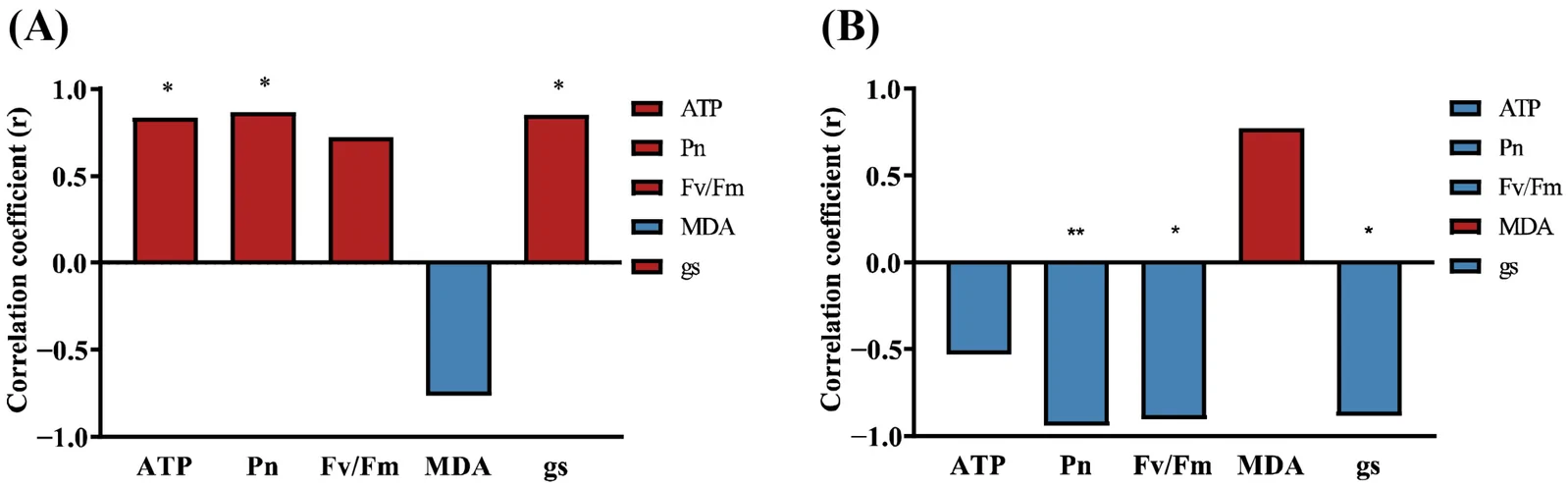
Analysis of the correlation between *MsndhB* RNA splicing efficiency and key physiological indicators under temperature stress conditions. (**A**,**B**) Pearson correlation coefficients between the RNA splicing efficiency of the *MsndhB* gene and key physiological indicators under high-temperature (**A**) and low-temperature (**B**) stress conditions. Each correlation was based on six observations (time-point means of three biological replicates). Because the data are derived from a time-course experiment, temporal autocorrelation may be present; the correlations should therefore be interpreted as exploratory. * *p* ≤ 0.05, ** *p* ≤ 0.01.

As shown in Figure 13, under high-temperature stress, splicing efficiency was significantly positively correlated with ATP content, Pn, and Gs (*p* ≤ 0.05), showed a positive but non-significant correlation with Fv/Fm, and negatively correlated with MDA content (not significant). Under low-temperature stress, the direction of correlations was reversed, splicing efficiency was strongly negatively correlated with Pn (*p* < 0.01), significantly negatively correlated with Fv/Fm and Gs (*p* ≤ 0.05), negatively correlated with ATP content, and positively correlated with MDA content, with the latter two not reaching statistical significance.

Collectively, these results indicated that *MsndhB* RNA splicing efficiency exhibited stress-specific associations with physiological parameters under temperature stress, with opposite correlation patterns observed between high- and low-temperature conditions.

## 3. Discussion

*MsndhB*, encoding a core subunit of the chloroplast NDH complex, may affect NDH-B accumulation and complex maturation through its RNA splicing efficiency, potentially contributing to photosynthetic homeostasis and temperature stress responses in alfalfa. In this study, the alfalfa cultivar *Medicago sativa* ‘Xinjiang Daye’ was used to systematically characterize the gene structure and sequence conservation of *MsndhB*, elucidate its RNA splicing response patterns to abiotic stresses, and reveal the associations between splicing efficiency and photosynthetic physiological parameters, thereby providing experimental evidence for understanding the post-transcriptional regulation of chloroplast genes in leguminous forage species.

In this study, the *MsndhB* gene was successfully cloned from alfalfa. The gene is 2167 bp in length and contains a 1479-bp coding sequence, and its intron boundaries conform to the conserved 5′-GT/3′-AC consensus sequence characteristic of chloroplast Group II introns [27,28]. The overall gene structure of *MsndhB* is highly consistent with that of *ndhB* genes reported in *Medicago* species and other higher plants [29]. Notably, this boundary pattern differs from the canonical GT-AG splice sites of the nuclear spliceosomal system [30] but represents characteristic structural feature of chloroplast Group II introns [9]. Phylogenetic analysis revealed that *MsndhB* clustered with other *Medicago* species in a highly supported clade, which is consistent with comparative genomic studies demonstrating that *M. falcata* and *M. sativa* exhibit high conservation in chloroplast genome structure, gene order, and gene content [29]. These findings further support the structural and evolutionary conservation of *ndhB* among legumes and angiosperms, and provide a basis for extrapolating *ndhB* splicing regulatory mechanisms characterised in tobacco, rice, and *Arabidopsis.* Previous studies have demonstrated that accurate splicing of the *ndhB* intron depends on nuclear-encoded RNA processing factors. For example, in *Arabidopsis*, nuclear-encoded factors such as EMB1270 and CFM2 specifically bind to the *ndhB* intron to facilitate its splicing [31]. Corresponding nuclear-encoded factors have also been reported to participate in *ndhB* splicing regulation in rice, maize, and other species [15,32,33], suggesting that the involvement of nuclear-encoded factors in chloroplast RNA splicing is conserved across different plant species.

The present study further reveals that temperature stress is the predominant environmental factor affecting *MsndhB* RNA splicing efficiency among all tested abiotic stresses. By contrast, under the current experimental conditions, salt stress and osmotic drought failed to trigger obvious alterations in splicing efficiency and only caused the downregulation of total transcript levels of this gene. These findings suggest that *MsndhB* RNA splicing may exhibit temperature-specific responsiveness. High -temperature treatment significantly suppressed *MsndhB* splicing, accompanied by the accumulation of unspliced pre-mRNA, a finding that corroborates previous observations in tobacco, *Arabidopsis*, and rice. Karcher and Bock [18] demonstrated that *ndhB* splicing is highly sensitive to high temperature in tobacco. Chloroplast transcriptome analysis by Castandet et al. [34] further revealed that high temperature globally reduces chloroplast RNA splicing and editing efficiencies. In rice, loss of function of the OsDYW2-MORF RNA processing complex impairs the splicing and editing of chloroplast genes, including *ndhB*, resulting in an albino and lethal phenotype in mutants under high-temperature conditions [35]. Collectively, these studies indicate that *ndhB* splicing represents an important post-transcriptional regulatory component involved in plant responses to high-temperature stress. Correspondingly, under high-temperature stress, Pn, Gs, Fv/Fm, and ATP content in alfalfa continuously declined with increasing stress duration, accompanied by photosynthetic pigment degradation, a progressive decrease in the chlorophyll a/b ratio, progressive MDA accumulation, and increased NPQ. The decrease in the chlorophyll a/b ratio indicates a relative increase in the proportion of chlorophyll b and thus an alteration in the composition of the photosynthetic pigments. Because chlorophyll b is predominantly associated with the light-harvesting complexes [36], this change may reflect a reorganization of the photosynthetic antenna system, thereby affecting the distribution of absorbed light energy [37]. Together with the decrease in total photosynthetic pigment content and the declines in Fv/Fm and Pn, these results suggest that high-temperature stress compromised the light-harvesting system and photosynthetic apparatus of alfalfa. Similar responses, including enhanced photoinhibition and increased membrane lipid peroxidation, have also been reported in tomato, grapevine, and wheat under high-temperature stress [38,39,40], suggesting that high-temperature-induced impairment of photosynthetic function is a common response across diverse plant species.

Considering the concurrent changes in photosynthetic performance and oxidative damage parameters under high-temperature stress in this study, the suppression of *MsndhB* splicing may be associated with impaired NDH complex-related functions. The NDH complex mediates cyclic electron flow around photosystem I and contributes to maintaining chloroplast electron transport balance, regulating the ATP/NADPH ratio, and protecting photosynthetic stability under stress conditions [20,21,41]. A high-temperature-induced reduction in *MsndhB* splicing efficiency could potentially reduce the production of mature *MsndhB* transcripts, which may affect NDH complex-associated functions and potentially contribute to disturbances in chloroplast energy metabolism and redox homeostasis. Such alterations might further contribute to reduced PSII photochemical efficiency and enhanced oxidative damage. It should be emphasized that these mechanistic inferences are derived from transcript-level data; whether the reduced splicing efficiency translates into altered NDH-B protein abundance, NDH complex abundance or activity, and NDH-dependent cyclic electron flow remains to be experimentally verified. Notably, a partial recovery of *MsndhB* splicing efficiency was observed at the later stage of stress, which may reflect a dynamic regulatory response of alfalfa to high-temperature stress; however, the underlying mechanisms remain to be elucidated.

In contrast to the effects of high temperature, low-temperature treatment significantly enhanced *MsndhB* splicing efficiency, with pre-mRNA continuously decreasing and mature mRNA abundance remaining relatively stable. Previous studies have shown that rice CDE4 plays an important role in maintaining the proper splicing of chloroplast transcripts, including *ndhB*, *ndhA*, and *rpl2*, under low-temperature conditions, and the *cde4* mutant exhibits splicing defects and a low-temperature-sensitive phenotype [25]. Similarly, the *Arabidopsis atrh3* mutant shows reduced *ndhB* splicing efficiency under low-temperature stress [26]. Notably, although both low- and high-temperature stresses resulted in decreases in Pn, Gs, Fv/Fm, and ATP content, accompanied by decreased photosynthetic pigment levels, a progressive decrease in the chlorophyll a/b ratio, accumulated MDA, and increased NPQ, the magnitude of these physiological responses and the underlying regulatory mechanisms differed between the two stress conditions. Compared with high-temperature stress, the changes in these physiological parameters under low-temperature treatment were relatively moderate, and Ci remained stable, suggesting that stomatal limitation may not be the primary factor in the low-temperature-induced decline in photosynthesis. Based on these observations, we speculate that low-temperature-induced enhancement of *MsndhB* splicing may contribute to maintaining NDH complex-associated functions, potentially partially alleviating the adverse effects of low temperature on the photosynthetic system, although this interpretation remains to be verified at the protein and complex levels. Nevertheless, although compensatory enhancement of NPQ could dissipate part of the excess excitation energy, it remained insufficient to fully overcome low-temperature-induced photosynthetic constraints, and was ultimately accompanied by decreased photosynthetic efficiency and enhanced membrane lipid peroxidation. A decrease in the chlorophyll a/b ratio was also observed under low-temperature stress, possibly suggesting a comparable alteration in the composition of the photosynthetic pigment pool, as discussed above for high-temperature stress.

Correlation analysis showed opposite association patterns under high- and low-temperature stresses. Because these data were derived from a time-course experiment, temporal autocorrelation may be present and the observations may not be fully independent. Therefore, the correlation results should be interpreted as covariation trends rather than evidence of causality. Under high-temperature stress, splicing efficiency was positively correlated with ATP content, Pn, and Gs, which is consistent with the inference that high-temperature-induced splicing suppression may affect NDH complex-associated functions. By contrast, under low-temperature stress, splicing efficiency showed a significant negative correlation with Pn, suggesting that photosynthetic impairment under low-temperature stress involves multiple regulatory processes [42], with RNA splicing representing one component of the overall response [42,43]. In other words, the potential beneficial effect of enhanced splicing on NDH function may have been insufficient to offset other low-temperature-induced inhibitory factors.

**Figure 14:**
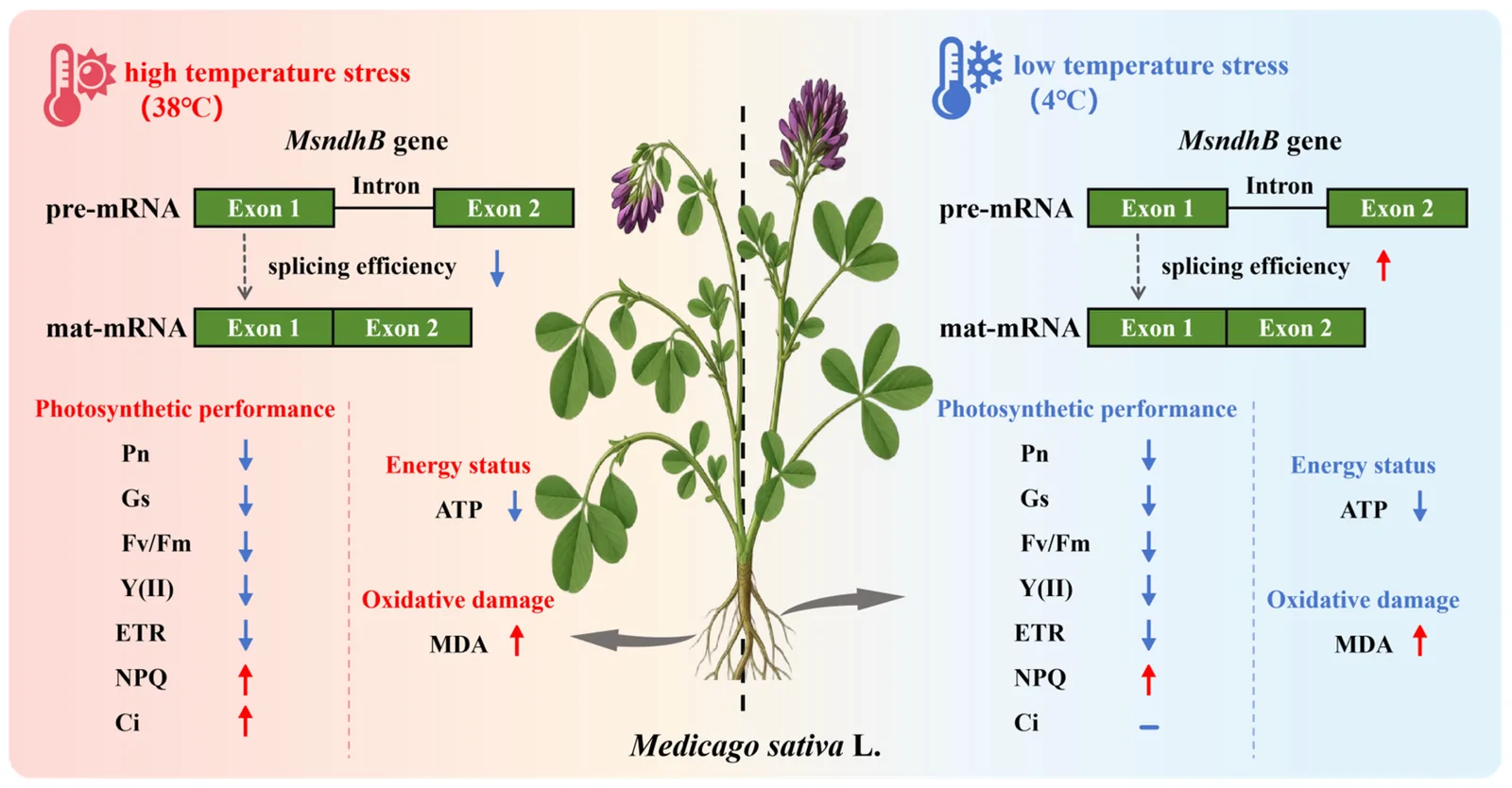
Proposed model illustrating temperature-responsive changes in *MsndhB* RNA splicing and their association with photosynthetic performance in *Medicago sativa* under high- and low-temperature stress.

Furthermore, the present study revealed that *MsndhB* splicing efficiency exhibited pronounced developmental stage- and tissue-dependent patterns. At the flowering stage, although the transcript levels of both pre-mRNA and mature mRNA reached relatively high levels, splicing efficiency was lower than that at the seedling and branching stages, displaying a “high transcription but low splicing” pattern. We speculate that during the flowering stage of alfalfa, the transcriptional output of *MsndhB* increases markedly, and that this elevated transcriptional output may exceed the processing capacity of the splicing machinery, such that the limited availability of nuclear-encoded PPR factors may partially constrain splicing efficiency. This interpretation is hypothetical and requires direct testing, for example, by quantifying the abundance of PPR splicing factors at different developmental stages. This possibility is consistent with the generally low-abundance expression of the PPR family in *Arabidopsis* [44]. Splicing efficiency in green tissues was significantly higher than that in roots, which may be associated with differences in plastid development and chloroplast RNA processing between green and non-green tissues-processes in which PPR proteins play important roles [45,46]. The high expression of rice CDE4 in young leaves and its direct involvement in *ndhB* splicing regulation [25] further support the concept that chloroplast RNA splicing is regulated in a developmental and tissue-dependent manner. As a perennial leguminous forage species, alfalfa undergoes repeated growth-flowering-pod development cycles each year, and the demand for photosynthetic products during flowering is relatively high compared with annual model plants. Therefore, the “high transcription but low splicing” pattern observed in this study may reflect a developmental response, whereby increased transcriptional output under conditions of limited splicing capacity could favor continued production of mature *MsndhB* transcripts during flowering. However, this interpretation remains speculative, and a limitation of this study is that NDH-B protein abundance, NDH complex abundance or activity, and NDH-dependent cyclic electron flow were not directly measured. Thus, the relationship between *MsndhB* splicing efficiency and NDH complex function remains to be experimentally verified. Based on these findings, we present a model summarizing changes in *MsndhB* RNA splicing and their associations with photosynthetic performance under high- and low-temperature stress (Figure 14).

Several unresolved questions remain after this study. A major limitation is that the relationship between *MsndhB* splicing changes and NDH-B protein abundance or NDH complex activity has not been directly examined. Protein-level detection and functional analyses of the NDH complex will therefore be important to clarify the consequences of altered splicing. In addition, the nuclear-encoded factors involved in regulating *MsndhB* splicing remain unidentified. Approaches such as RNA pull-down, yeast three-hybrid, and EMSA could be employed to screen potential regulatory factors [25,47,48,49], and future studies using these strategies will help elucidate the regulatory network underlying *MsndhB* RNA splicing. Furthermore, the possible interaction between *ndhB* RNA editing and splicing during temperature adaptation was not addressed in the present study and represents an interesting direction for future investigation.

## 4. Materials and Methods

### 4.1. Plant Materials and Growth Conditions

The alfalfa cultivar *Medicago sativa* ‘Xinjiang Daye’ was used in this study. Seeds were provided by the College of Grassland Science, Xinjiang Agricultural University. Seeds were surface-sterilized and germinated on moist filter paper in a light incubator under conditions of 25 °C, a 16-h light/8-h dark photoperiod, and a light intensity of 200 μmol·m^−2^·s^−1^. After 5–7 days of germination, seedlings of uniform growth were selected and transferred to a climate-controlled growth chamber for hydroponic culture. The seedlings were provided with half-strength Hoagland nutrient solution every 3 days. After 30 days of cultivation, uniformly grown seedlings were selected for subsequent abiotic stress treatments and expression analyses.

### 4.2. Abiotic Stress Treatments

The following treatments were administered to thirty-day-old seedlings: (1) osmotic stress, which was created by augmenting the nutrient solution with 20% (*w*/*v*) PEG-6000; (2) salt stress, imposed by supplementing the nutrient solution with 200 mM NaCl; (3) low-temperature stress, by transferring seedlings to a growth chamber at 4 °C (16-h light/8-h dark); and (4) high-temperature stress, which was achieved by transferring seedlings to a growth chamber at 38 °C (16-h light/8-h dark). Throughout the experiment, a separate set of seedlings maintained under normal growth conditions (25 °C, 16 h light/8 h dark) served as the untreated control. The second and third fully expanded leaves were collected at 0, 3, 6, 12, 24, and 48 h after treatment initiation. For each treatment and time point, three independent biological replicates were collected, each corresponding to one independent hydroponic container. For each replicate, leaves were randomly collected from multiple seedlings within the same container (approximately 15 seedlings per container) and pooled into a single sample before RNA extraction. All samples were immediately frozen in liquid nitrogen and stored at −80 °C until further use.

### 4.3. Sampling at Different Developmental Stages and Tissues

For soil-grown plants, apical leaves were collected at the seedling, branching, flowering, and pod-setting stages. At the early flowering stage, roots, stems, leaves, flower buds, and flowers were collected; at the pod-setting stage, pods were collected. All samples were immediately frozen in liquid nitrogen and stored at a temperature of −80 °C.

### 4.4. Genomic DNA and Total RNA Extraction

Genomic DNA was extracted using the Plant Genomic DNA Extraction Kit (Tiangen Biotech, Beijing, China) according to the manufacturer’s instructions. Total RNA was extracted from frozen samples using TRIzol reagent (Thermo Fisher Scientific, Waltham, MA, USA). RNA concentration and purity were assessed using a spectrophotometer (Thermo Fisher Scientific, Waltham, MA, USA), and samples with A_260_/A_280_ ratios of 1.8–2.1 were used for subsequent analyses.

### 4.5. Cloning and Sequence Analysis of the MsndhB

The NovoScript Plus All-in-one 1st Strand cDNA Synthesis SuperMix was employed to synthesise first-strand cDNA. The reference genome sequence of alfalfa *ndhB* (GenBank accession No. NC_042841.1) was used to design gene-specific primers for *MsndhB* using Primer Premier 5.0. The primers were synthesised by Xinjiang Youkang Biotechnology Co., Ltd., Urumqi, China; the sequences are listed in Supplementary Table S1. The PCR reaction mixture (25 μL) was composed of 12.5 μL of 2× Taq PCR Master Mix, 1 μL of forward and reverse primers (10 μM), 1 μL of cDNA template, and 9.5 μL of ddH_2_O. The amplification programme was as follows: initial denaturation at 95 °C for 3 min, followed by 35 cycles of denaturation at 95 °C for 15 s, annealing at 58 °C for 30 s, and extension at 72 °C for 1 min; and a final extension at 72 °C for 5 min. The PCR products were purified by gel extraction, separated by 1% agarose gel electrophoresis, and ligated into the pMD19-T vector (TaKaRa, Dalian, China). Escherichia coli DH5α competent cells were used to transform the recombinant plasmids. Positive clones were identified and subsequently submitted for Sanger sequencing verification. The nucleotide variation sites were identified by aligning the *MsndhB* sequence with the reference genome sequence using DNAMAN (version 9.0).

### 4.6. Bioinformatics Analysis

#### 4.6.1. Prediction of Physicochemical Properties and Conserved Domains

The molecular weight, theoretical isoelectric point (pI), instability index, aliphatic index, grand average of hydropathicity (GRAVY), and amino acid composition of the MsNDHB protein were predicted using the ProtParam tool (https://web.expasy.org/protparam/ (accessed on 12 May 2025)). Conserved domains were identified using the NCBI Batch CD-Search tool (https://www.ncbi.nlm.nih.gov/Structure/bwrpsb/bwrpsb.cgi (accessed on 11 May 2025)).

#### 4.6.2. Protein Structure Prediction

The protein sequence was submitted to the NPS@: SOPMA secondary structure prediction server (IBCP; https://npsa.lyon.inserm.fr/cgi-bin/npsa_automat.pl?page=npsa_sopma.html (accessed on 25 May 2025)) for secondary structure analysis. The protein sequence was subsequently submitted to SwissModel (Swiss Bioinformatics Resource Portal; https://www.swissmodel.expasy.org/ (accessed on 27 May 2025)) for three-dimensional structure prediction [50].

#### 4.6.3. Phylogenetic Analysis

NDHB protein sequences from 17 representative species, including members of Leguminosae, Poaceae, gymnosperms, and cyanobacteria, were obtained from the NCBI database (https://www.ncbi.nlm.nih.gov/ (accessed on 9 June 2025)). Sequence alignment and phylogenetic analysis were performed using MEGA7 (version 7.0.26) based on the Neighbor-Joining method. The reliability of the phylogenetic tree was assessed by bootstrap analysis with 1000 replicates [51,52].

#### 4.6.4. Amino Acid Motif and Gene Structure Analysis

Protein motif analysis was conducted using the MEME Suite online platform (http://meme-suite.org/ (accessed on 15 June 2025)) with the maximum number of motifs set to 10. Gene structure analysis was performed using GSDS 2.0 (http://gsds.gao-lab.org/ (accessed on 16 June 2025)) to generate exon–intron organization diagrams.

#### 4.6.5. Analysis of Splice Sites Across Multiple Species

Full-length genomic sequences and coding sequences (CDS) of chloroplast *ndhB* genes from different species (including Vicia, Uraria, cyanobacteria, etc.) were retrieved from the NCBI database. Multiple sequence alignments were performed using DNAMAN (version 9.0), and the 5′ and 3′ splice site sequences were manually annotated. The intron type was determined based on conserved intron sequence features.

### 4.7. Analysis of RNA Splicing Efficiency

Chloroplast RNA splicing was analyzed by RT-PCR and RT-qPCR using gene-specific primers as described by de Longevialle et al. [53] and Huang et al. [54]. For endpoint RT-PCR analysis of RNA splicing efficiency, chloroplast genes were amplified with specific primers flanking the introns. Primers were synthesized by Xinjiang Youkang Biotechnology Co., Ltd. (Urumqi, China), and their sequences are listed in Supplementary Table S2.

For qRT-PCR analysis of RNA splicing efficiency, specific primers were designed to amplify the intron–exon boundary (for pre-mRNA, designated as U) and the exon–exon junction (for mature mRNA, designated as S). The amplification efficiencies of the two primer pairs were evaluated using standard curves generated from serial dilutions of cDNA. The pre-mRNA primer pair showed an amplification efficiency of 95.1% (slope = −3.4446, R^2^ = 0.9928), and the mature mRNA primer pair showed an amplification efficiency of 93.0% (slope = −3.5023, R^2^ = 0.9903). Because the two primer pairs showed comparable amplification efficiencies (difference < 5%), the relative transcript abundances of S and U were quantified using the 2^−ΔCt^ method. *MsActin* (AA660796) was used as the internal reference gene. ΔCt was calculated as Ct(target transcript) − Ct(*MsActin*), and the normalized relative quantities of S and U transcripts were obtained before calculating splicing efficiency. Splicing efficiency was defined as the percentage of mature mRNA in the total *MsndhB* transcript pool and was calculated using the following equation: splicing efficiency (%) = [S/(S + U)] × 100, where S and U represent the relative quantities of mature mRNA and unspliced pre-mRNA, respectively, as determined using the 2^−ΔCt^ method. The fold changes of S and U transcripts relative to the 0 h time point under different stress treatments were analyzed using the 2^−ΔΔCt^ method. All primer sequences were synthesized by Xinjiang Youkang Biotechnology Co., Ltd. (Urumqi, China) and deposited in Supplementary Table S2.

### 4.8. Measurement of Physiological Parameters

#### 4.8.1. Photosynthetic Pigment Content Determination

Chlorophyll content was determined according to the method described by Wu et al. [55]. Approximately 0.1 g of the second fully expanded leaf was cut into pieces in a 10 mL centrifuge tube containing 5 mL of 95% ethanol, and shaken until all leaf pieces settled at the bottom of the tube. The tube was then kept at room temperature in the dark until the leaves were completely decolorized. The absorbance of the extract was measured at 665, 649 and 470 nm. The following equations were used for calculation:

Chl a content (mg/g) = [(13.95 × OD665 − 6.88 × OD649) × V]/(1000 × W)

Chl b content (mg/g) = [(24.96 × OD649 − 7.32 × OD665) × V]/(1000 × W)

Car (mg/g) = (1000 × OD470 − 2.05 × Ca − 114.8 × Cb)/245

where OD represents the absorbance at the corresponding wavelength; V is the total volume of the chlorophyll extraction supernatant (mL); W is the fresh weight of the leaf (g). If the sample was diluted before measurement, the dilution factor should be included in the calculation [56].

#### 4.8.2. Photosynthetic Gas-Exchange Parameter Measurement

Net photosynthetic rate (Pn), stomatal conductance (Gs), intercellular CO_2_ concentration (Ci), and transpiration rate (E) were measured using a Li-6400XT portable photosynthesis system (LI-COR, Lincoln, NE, USA). Measurements were performed under the following conditions: photosynthetically active photon flux density of 1000 μmol·m^−2^·s^−1^, CO_2_ concentration of 400 μmol·mol^−1^, leaf chamber temperature of 25 °C, and relative humidity of 60–70%.

#### 4.8.3. Chlorophyll Fluorescence Parameter Measurement

Chlorophyll fluorescence parameters were determined using a portable modulated chlorophyll fluorometer (MINI-PAM-II, Walz, Effeltrich, Germany) following the manufacturer’s instructions. After dark adaptation for 30 min, the maximum quantum efficiency of photosystem II (Fv/Fm), actual photochemical quantum yield (Y(II)), relative electron transport rate (ETR), and non-photochemical quenching (NPQ) were measured.

#### 4.8.4. Determination of Malondialdehyde and ATP Contents

The MDA and ATP contents were determined using commercial assay kits (MDA: BC0020; ATP: BC0300; Solarbio, Beijing, China) according to the manufacturer’s instructions. Briefly, approximately 0.1 g of fresh leaf tissue was homogenized in 1 mL of ice-cold extraction buffer. For MDA determination, absorbance was measured at 532 and 600 nm after the thiobarbituric acid reaction. Blank-corrected absorbance values were calculated as ΔA532 = A532(sample) − A532(blank) and ΔA600 = A600(sample) − A600(blank), and ΔA was calculated as ΔA532 − ΔA600. MDA content was then calculated as MDA (nmol g^−1^ FW) = 32.258 × ΔA × F/W, where W is the fresh weight of the sample (g) and F is the dilution factor. For ATP determination, absorbance was measured at 340 nm using the hexokinase-glucose-6-phosphate dehydrogenase method. A 0.625 μmol mL^−1^ ATP standard was used for calibration, and the absorbance change was calculated as ΔA = A2 − A1. ATP content was calculated as ATP (μmol g^−1^ FW) = 0.625 × ΔA_sample/(ΔA_standard × W), where W is the fresh weight of the sample (g).

### 4.9. Correlation Analysis

Pearson bivariate correlation analysis was performed using SPSS 25.0 software to evaluate the relationships between *MsndhB* RNA splicing efficiency and the physiological parameters (Pn, Gs, Fv/Fm, ATP, MDA) under temperature stress. Pearson correlation coefficients between *MsndhB* splicing efficiency and each physiological parameter were calculated using the mean values of the three biological replicates at each time point (*n* = 6 per treatment). Correlation coefficients (r) and significance levels (*p*-values) were calculated. Graphs were generated using GraphPad Prism 9.0 software.

### 4.10. Statistical Analysis

All quantitative data are presented as mean ± standard deviation (SD) from three independent biological replicates. Statistical analyses were conducted using SPSS 25.0 software. Differences among groups were assessed by one-way analysis of variance (ANOVA) followed by Tukey’s HSD post hoc multiple comparison test; for the time-course data, the six time points within each treatment were compared separately by one-way ANOVA. A *p*-value < 0.05 was considered statistically significant. Data visualization and graphing were performed using GraphPad Prism 9.0 software (GraphPad Software, San Diego, CA, USA).

## 5. Conclusions

In summary, our results show that *MsndhB* RNA splicing in alfalfa responds differently to the abiotic stresses tested. Temperature stress appears to be the key factor affecting splicing: high temperature suppressed *MsndhB* splicing, whereas low temperature promoted it, while salt and drought mainly affected transcript levels. The correlations between splicing efficiency and photosynthetic parameters also differed between high- and low-temperature stress. These results suggest a potential role of *MsndhB* RNA splicing in chloroplast responses to temperature stress. These findings add to our understanding of post-transcriptional regulation of chloroplast genes under environmental stress in leguminous forages and provide a basis for future work to identify the regulators of *MsndhB* splicing and to explore the temperature-responsive mechanisms involved.

## Figures and Tables

**Table 1:** Predicted splice sites in *ndhB* mRNA.

Gene Symbol	5′SS	3′SS	Intron Type	Note
*MsndhB*	GT	AC	Group II	-
*MfndhB*	GT	AC	Group II	-
*MvndhB*	GT	AC	Group II	-
*MtndhB*	GT	AC	Group II	-
*CandhB*	GT	AC	Group II	-
*VkndhB*	GA	AG	Group II	-
*AhndhB*	GT	AC	Group II	-
*AtndhB*	GT	AC	Group II	-
*GmndhB*	GT	AC	Group II	-
*UcndhB*	GT	AG	Group II	-
*TandhB*	GT	AC	Group II	-
*OsndhB*	GT	AC	Group II	-
*ZmndhB*	GT	AC	Group II	-
*HandhB*	GT	AC	Group II	-
*GbndhB*	GT	AC	Group II	-
*MpndhB*	GT	AC	Group II	-
*SspndhB*	-	-	-	No introns

## Data Availability

The cloned *MsndhB* sequence has been deposited in the NCBI GenBank database under accession number [QB044729]. The original contributions presented in this study are included in the article/Supplementary Materials. Further inquiries can be directed to the corresponding author.

## Supplementary Materials

The following supporting information can be downloaded at: https://www.mdpi.com/article/10.3390/plants15203198/s1. Table S1: Primers used for gene cloning; Table S2: Primers used for RT-PCR and qRT-PCR; Figure S1: Alignment of ‘Xinjiang Daye’ alfalfa with reference sequences; Table S3: Prediction of the primary structure of the MsNDHB protein; Table S4: Amino acid composition analysis of MsNDHB; Table S5: Prediction of the secondary structure of NDHB proteins from different species; Table S6: Model quality parameters of the 17 NDHB proteins predicted by SWISS-MODEL; Figure S2: Changes in physiological parameters of *Medicago sativa* ‘Xinjiang Daye’ under salt stress. Thirty-day-old seedlings grown hydroponically were subjected to salt stress (200 mM NaCl) for 0, 3, 6, 12, 24 and 48 h. (A–H) Total chlorophyll content, ATP content, Pn, Ci, Gs, E, MDA content and maximum quantum efficiency of PSII (Fv/Fm), respectively. Values are presented as means ± SD of three independent experiments (*n* = 3). Different lowercase letters indicate significant differences among treatment time points (*p* ≤ 0.05; one-way ANOVA followed by Tukey’s multiple comparison test); Figure S3: Changes in physiological parameters of *Medicago sativa* ‘Xinjiang Daye’ under drought stress. Thirty-day-old seedlings grown hydroponically were subjected to drought stress (20% PEG-6000) for 0, 3, 6, 12, 24 and 48 h. (A–H) Total chlorophyll content, ATP content, Pn, Ci, Gs, E, MDA content and maximum quantum efficiency of PSII (Fv/Fm), respectively. Values are presented as means ± SD of three independent experiments (*n* = 3). Different lowercase letters indicate significant differences among treatment time points (*p* ≤ 0.05; one-way ANOVA followed by Tukey’s multiple comparison test); Table S7: NDH-B protein sequences of the 17 species used for phylogenetic analysis in Figure 2.
