# Supplementary material for: RNA Splicing of the MsndhB Gene and Photosynthetic Characteristics in Alfalfa (Medicago sativa) Under High- and Low-Temperature Stress
Source: Plants (Basel). 2026 Oct 18;15(20):3198. doi: 10.3390/plants15203198 (PMC13708757; doi:10.3390/plants15203198)
Supplement: Supplementary file 1 [file plants-15-03198-s001.zip › Appendix-revise.pdf]

Table S1 Primers used for gene cloning

Primer Name	Sequence (5′→3′)
*MsndhB*F	AGAACACGGGGGACGAGCTCATGAAAGCCTTTCATTTGCTTCTTTTC
*MsndhB*R	ACCATGGTGTCGACTCTAGAAAAAAGGGTATCCTGAGCAATTTC

Table S2 Primers used for RT-PCR and qRT-PCR

Primer Name	Sequence (5′→3′)
MsndhB-1F	GCCTTTCATTTGCTTCTTTTCG
MsndhB-1R	CTGCTTGCCATCCACACCA
MsndhB-preF	TCCTGACGTATATGAAGGAGTGC
MsndhB-preR	ACCGGGATTCATTGTTCCTGA
MsndhB-matF	ATATGAAGGATCTCCGACTCCAG
MsndhB-matR	CTTGCTTATTCGTCCATAGGTCAA

Note: *MsndhB*-1: Primers for PCR amplification; *MsndhB*-pre and *MsndhB*-mat: Primers used for the amplification of pre-mRNA and mat-mRNA in qRT-PCR, respectively.

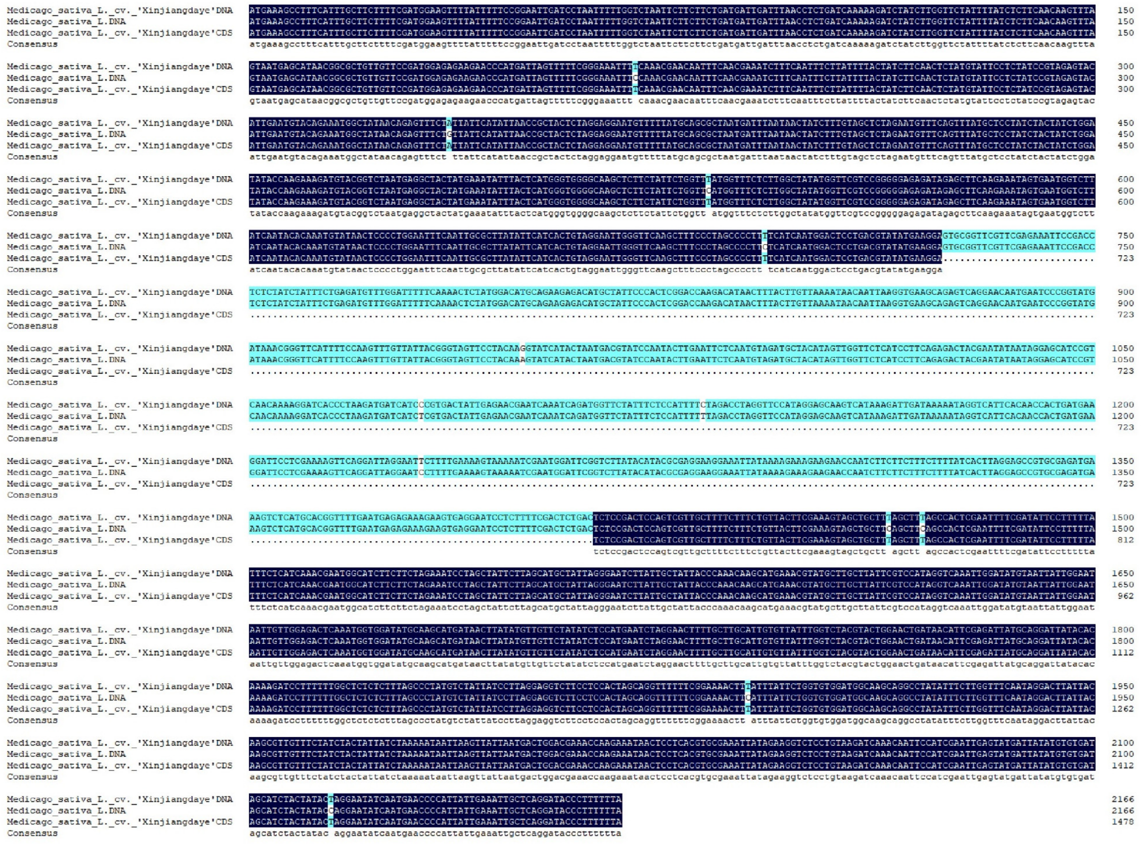

Figure S1 Alignment of 'Xinjiang Daye' alfalfa with reference sequences

Table S3 Prediction and analysis of the primary structure of the protein encoded by the MsNDHB

Primary structure characteristics	MsNDHB
Number of amino acids	492
Isoelectric point (pI)	5.11
Molecular weight (Mw)	54695.56
Molecular formula	$C_{2550}H_{3953}N_{585}O_{689}S_{27}$
Positively charged residues	24
Negatively charged residues	32
Grand average of hydropathicity (GRAVY)	0.737
Aliphatic index (AI)	121.71
Instability index (II)	41.84
Theoretical half-life	30

Table S4 Amino acid composition analysis of MsNDHB

Amino Acid	Number	Percentage (%)	Amino Acid	Number	Percentage (%)
Ala（A）	27	5.5	Met（M）	18	3.7
Arg（R）	12	2.4	Phe（F）	35	7.1
Asn（N）	19	3.9	Pro（P）	15	3.0
Asp（D）	13	2.6	Ser（S）	49	10.0
Cys（C）	9	1.8	Thr（T）	30	6.1
Gln（Q）	11	2.2	Trp（W）	7	1.4
Glu（E）	19	3.9	Tyr（Y）	23	4.7
Gly（G）	37	7.5	Val（V）	21	4.3
His（H）	4	0.8	Pyl（O）	0	0.0
Ile（I）	56	11.4	Sec（U）	0	0.0
Leu（L）	75	15.2	Lys（K）	12	2.4

Table S5 Prediction of the secondary structure of NDHB proteins from different species

Protein name	Alpha helix%	Extended strand%	Beta turn%	Random coil%
MsNDHB	49.8	17.89	4.67	27.64
MfNDHB	49.8	17.89	4.67	27.64
MvNDHB	49.8	17.89	4.67	27.64
MtNDHB	49.8	17.89	4.67	27.64
CaNDHB	49.02	19.22	4.51	27.25
VkNDHB	48.11	17.30	4.37	30.22
AhNDHB	50.09	17.20	6.43	26.28
AtNDHB	49.39	17.48	4.88	28.25
GmNDHB	52.44	15.85	5.69	26.02
UcNDHB	50.70	17.37	6.39	25.55
TaNDHB	50.86	17.27	5.57	26.30
OsNDHB	52.35	16.47	4.90	26.27
ZmNDHB	52.16	15.69	4.71	27.45
HaNDHB	48.82	17.84	5.69	27.65
GbNDHB	50.21	17.23	4.68	27.87
MpNDHB	48.70	17.56	5.79	27.94

SSpNDHB	57.39	12.45	6.03	24.12

Table S6 Model quality parameters of the 17 NDHB proteins predicted by SWISS-MODEL.

**Protein name**	Template PDB ID	Template description	Sequence identity (%)	Coverage (%)	GM QE	Ramachandran favoured (%)	MolProbity score
MsNDHB	A0A4P8F3R5.1. A	AlphaFold DB model	99.19	100	0.9	97.35	1.08
MfNDHB	A0A4P8F3R5.1. A	AlphaFold DB model	99.19	100	0.9	97.35	1.08
MvNDHB	A0A4P8F3R5.1. A	AlphaFold DB model	99.19	100	0.9	97.35	1.08
MtNDHB	A0A4P8F3R5.1. A	AlphaFold DB model	98.58	100	0.9	97.35	1.08
CaNDHB	A0A7T3QNX6.1. A	AlphaFold DB model	95.29	100	0.88	97.05	1.21
VkNDHB	A0A4P8F3R5.1.A	AlphaFold DB model	97.36	98	0.88	95.21	1.53
AhNDHB	7wff.1. B	NAD(P)H-quinone oxidoreductase subunit 2, chloroplastic (PDB)	94.63	95	0.85	89.33	1.63
AtNDHB	A0A223A792.1. A	AlphaFold DB model	99.19	100	0.9	97.35	0.89
GmNDHB	A0A890CI70.1. A	AlphaFold DB model	98.37	100	0.9	97.14	1.12
UcNDHB	A0A890CI70.1. A	AlphaFold DB model	98.58	98	0.88	94.39	1.43
TaNDHB	A0A6M3SBQ4.1. A	AlphaFold DB model	97.84	98	0.87	96.15	1.30
OsNDHB	A0A1W5HQ00.1. A	AlphaFold DB model	100	100	0.89	97.24	0.91
ZmNDHB	A0A513WYK4.1. A	AlphaFold DB model	99.8	100	0.89	96.85	0.98
HaNDHB	A0A7U0KTF5.1. A	AlphaFold DB model	100	100	0.88	97.44	0.92
GbNDHB	A0A650DDG7.1. A	AlphaFold DB model	92.98	100	0.91	98.29	1.09
MpNDHB	P06257.1. A	AlphaFold DB model	100	100	0.9	98.20	0.96
SSpNDHB	A0A7C3PRF0.1. A	AlphaFold DB model	80.3	99	0.93	97.85	1.09

Note: Template 7wff.1.B is an experimental cryo-EM structure from the Protein Data Bank (PDB); all other templates are models from the AlphaFold Database (AlphaFold DB).

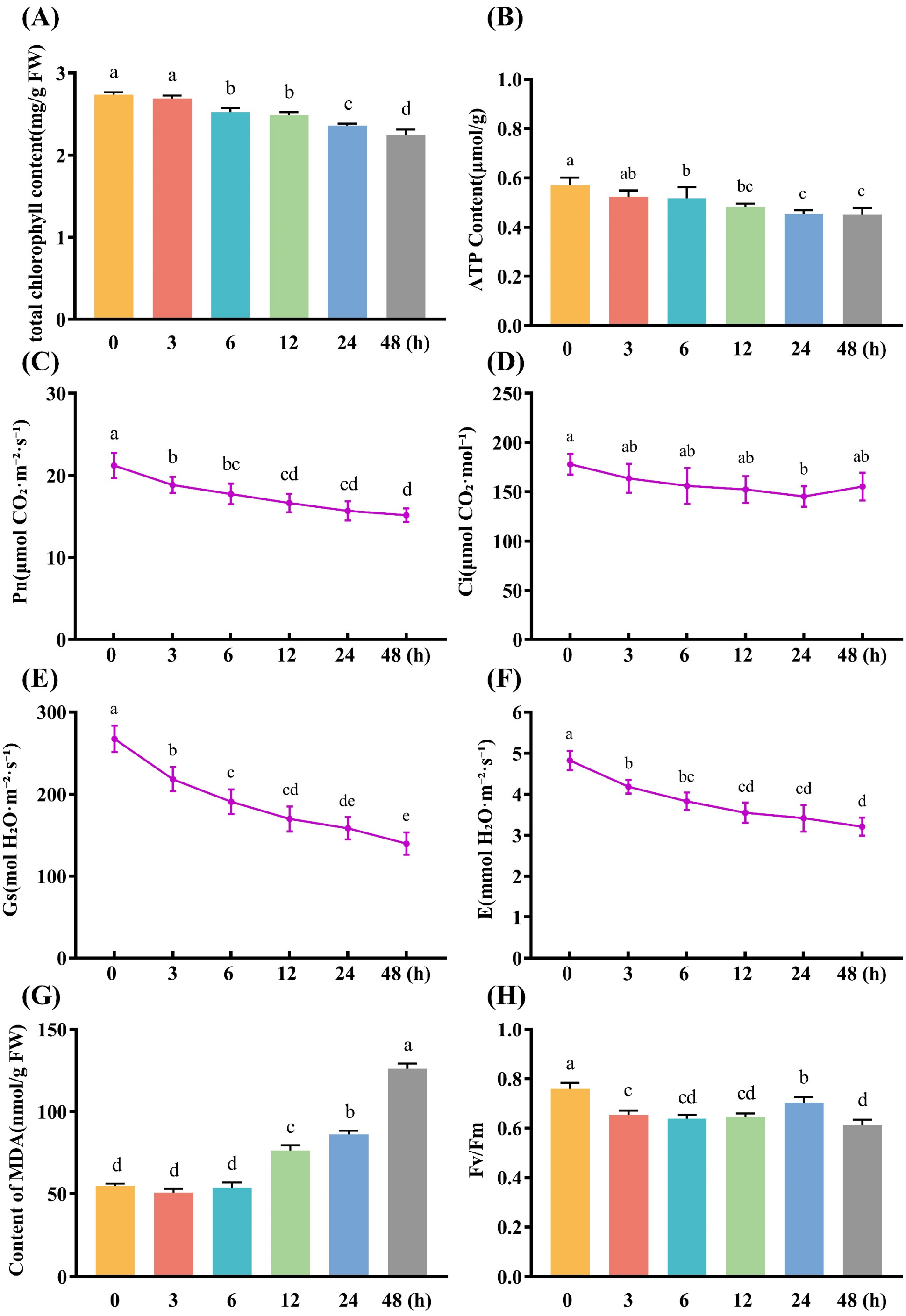

Figure S2 Changes in physiological parameters of *Medicago sativa* 'Xinjiang Daye' under salt stress. Thirty-day-old seedlings grown hydroponically were subjected to salt stress (200 mM NaCl) for 0, 3, 6, 12, 24 and 48 h. (A-H) Total chlorophyll content, ATP content, Pn, Ci, Gs, E, MDA content and maximum quantum efficiency of PSII (Fv/Fm), respectively. Values are presented as means ± SD of three independent experiments (n = 3). Different lowercase letters indicate significant differences among treatment time points ($P \leq 0.05$; one-way ANOVA followed by Tukey's multiple comparison test).

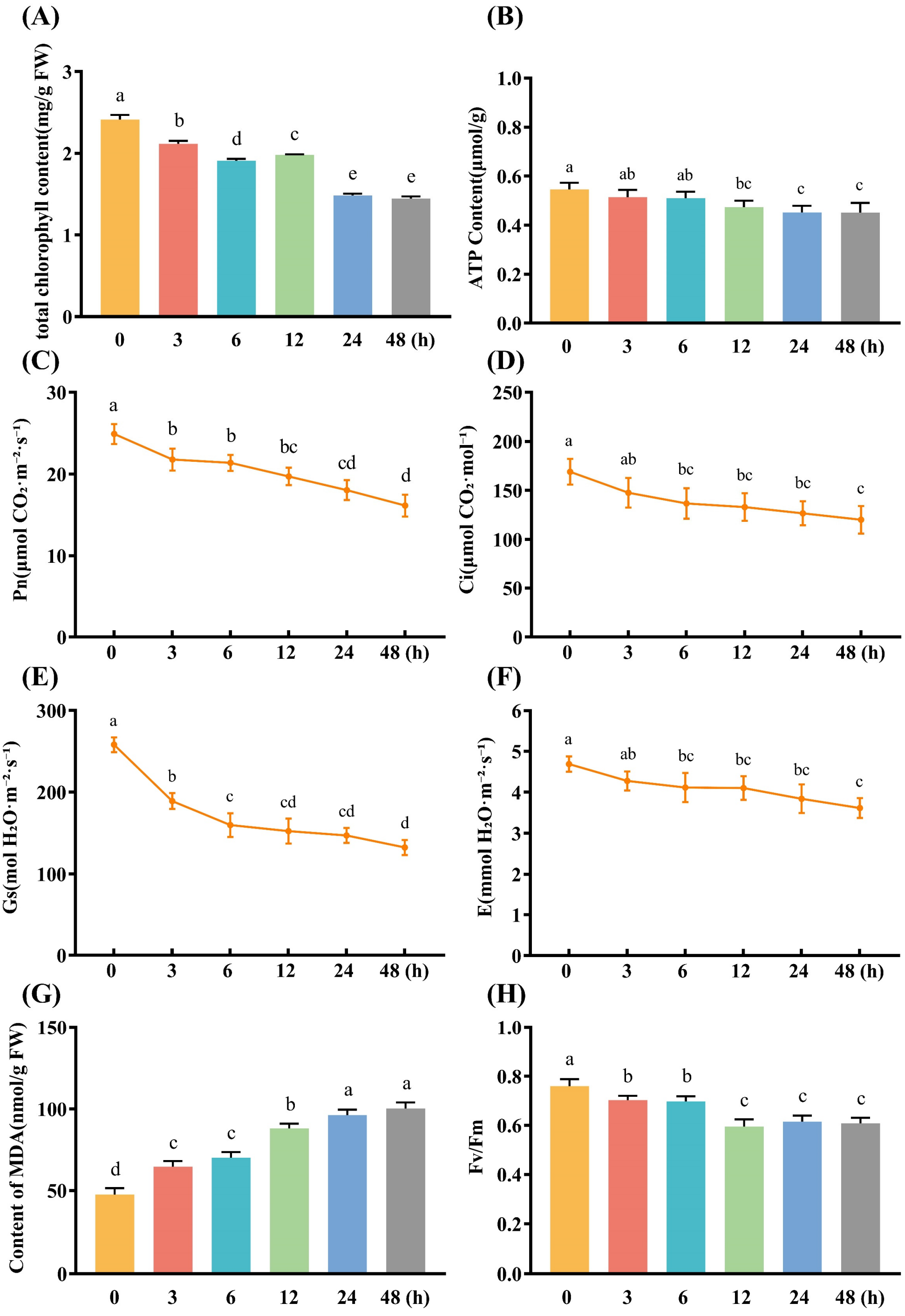

Figure S3 Changes in physiological parameters of *Medicago sativa* 'Xinjiang Daye' under drought stress. Thirty-day-old seedlings grown hydroponically were subjected to drought stress (20% PEG-6000) for 0, 3, 6, 12, 24 and 48 h. (A-H) Total chlorophyll content, ATP content, Pn, Ci, Gs, E, MDA content and maximum quantum efficiency of PSII (Fv/Fm), respectively. Values are presented as means ± SD of three independent experiments (n = 3). Different lowercase letters indicate significant differences among treatment time points ($P \leq 0.05$; one-way ANOVA followed by Tukey's multiple comparison test).
